# Identification of hub genes and potential molecular mechanisms related to radiotherapy sensitivity in rectal cancer based on multiple datasets

**DOI:** 10.1186/s12967-023-04029-2

**Published:** 2023-03-06

**Authors:** Pengfei Zhao, Hongchao Zhen, Hong Zhao, Yongjie Huang, Bangwei Cao

**Affiliations:** 1 Department of Radiotherapy, Beijing Friendship Hospital, Capital Medical University, Beijing, 100050 P.R. China; 2 Department of Oncology, Beijing Friendship Hospital, Capital Medical University, No.95 Yong An Road, Xicheng District, Beijing, 100050 P.R. China

**Keywords:** READ, Radiotherapy sensitivity, Hub genes, Molecular mechanisms, Regulatory networks

## Abstract

**Background:**

Radiotherapy resistance is the main cause of low tumor regression for locally advanced rectum adenocarcinoma (READ). The biomarkers correlated to radiotherapy sensitivity and potential molecular mechanisms have not been completely elucidated.

**Methods:**

A mRNA expression profile and a gene expression dataset of READ (GSE35452) were acquired from The Cancer Genome Atlas (TCGA) and Gene Expression Omnibus (GEO) databases. Differentially expressed genes (DEGs) between radiotherapy responder and non-responder of READ were screened out. Gene ontology (GO) analysis and Kyoto Encyclopedia of Genes and Genomes (KEGG) pathway analysis for DEGs were performed. Random survival forest analysis was used to identified hub genes by randomForestSRC package. Based on CIBERSORT algorithm, Genomics of Drug Sensitivity in Cancer (GDSC) database, Gene set variation analysis (GSVA), enrichment analysis (GSEA), nomogram, motif enrichment and non-coding RNA network analyses, the associations between hub genes and immune cell infiltration, drug sensitivity, specific signaling pathways, prognosis prediction and TF – miRNA regulatory and ceRNA network were investigated. The expressions of hub genes in clinical samples were displayed with the online Human Protein Atlas (HPA).

**Results:**

In total, 544 up-regulated and 575 down-regulated DEGs in READ were enrolled. Among that, three hubs including *PLAGL2*, *ZNF337* and *ALG10* were identified. These three hub genes were significantly associated with tumor immune infiltration, different immune-related genes and sensitivity of chemotherapeutic drugs. Also, they were correlated with the expression of various disease-related genes. In addition, GSVA and GSEA analysis revealed that different expression levels of *PLAGL2*, *ZNF337* and *ALG10* affected various signaling pathways related to disease progression. A nomogram and calibration curves based on three hub genes showed excellent prognosis predictive performance. And then, a regulatory network of transcription factor (*ZBTB6*) - mRNA (*PLAGL2*) and a ceRNA network of miRNA (has-miR-133b) - lncRNA were established. Finally, the results from HPA online database demonstrated the protein expression levels of PLAGL2, ZNF337 and ALG10 varied widely in READ patients.

**Conclusion:**

These findings indicated that up-regulation of *PLAGL2*, *ZNF337* and *ALG10* in READ associated with radiotherapy response and involved in multiple process of cellular biology in tumor. They might be potential predictive biomarkers for radiotherapy sensitivity and prognosis for READ.

**Supplementary Information:**

The online version contains supplementary material available at 10.1186/s12967-023-04029-2.

## Background

Colorectal cancer is one of the most common digestive system malignancies. New epidemiological data demonstrates that colorectal cancer is the third most common cancer and the third leading cause of cancer death in both men and women in 2022 globally. Among these cases, nearly one-third are rectum adenocarcinoma (READ) [1]. Preoperative chemoradiotherapy (CRT) or combined with total neoadjuvant chemotherapy (TNT) followed by total mesorectal excision is recommended as the standard strategy for locally advanced READ patients with T3-4, node-negative/positive and no distant metastasis. The locoregional recurrence was significantly reduced and decreased to 5−9% with the application of multidisciplinary treatment [2, 3]. However, distant metastasis was the major cause of treatment failure and the 5-year distant metastasis rate was as high as about 30% [4, 5]. Pathological complete response (pCR) after neoadjuvant CRT was associated with better long-term survival outcome, such as disease-free survival (DFS) and overall survival (OS). Nevertheless, the pCR rate was only between 15% and 20% in READ patients administered neoadjuvant CRT [6–8]. So far, various efforts have been tried to search sensitive, specific and accurate biomarkers to predict the response of READ to radiotherapy, especially the pCR status. However, none has been applied successfully in clinic except the traditional but effective prognosis prediction methods of tumor-node-metastasis (TNM) staging system.

Radiotherapy can cause lethal lesions to tumor cells by damaging DNA double-strand breaks (DSB) directly or by generation of free radicals and reactive oxygen species indirectly induced by its ionizing radiation. The radio-resistance of tumor cells is the major obstacle for radiotherapy application. Previous study had shown that PI3K/Akt signaling and activation of Akt1 might be involved in irradiation resistance by accelerating the repair of DNA-DSB [9, 10]. Moreover, other DNA damage response signaling pathways including the activation of DNA damage sensing, early transduction pathways and cell cycle arrest were associated with tumor radio-resistance [11]. Some pathological features and molecular biomarkers including DNA mutation and DNA methylation, gene expression profiles, proteins and metabolites, tumor immune microenvironment and several microRNAs had the potential to predict the efficacy of preoperative CRT [12]. However, majority of the biomarkers were lack of sensitivity and specificity.

Anticancer immunotherapies have revolutionized cancer treatment in recent years. Radiotherapy can enhance the effect of immune checkpoint inhibitors by increasing CD8 + T-cell infiltration of tumors, increasing the recognition of host immune system to tumors, increasing the clearance of antigen presenting cells to tumors and remodeling the tumor immune microenvironment [13]. Thus, the combination of radiotherapy and immunotherapy can convert an immune cold tumor to a hot tumor by boosting antitumor immunity. The phase III PACIFIC trial showed that consolidation durvalumab (an anti-programmed death ligand-1 antibody) after concurrent chemoradiotherapy had robust and sustained OS and durable PFS benefit compared with the placebo in stage III non-small-cell lung cancer [14]. Similarly, CRT combined with immunotherapy as neoadjuvant therapy could obtain higher pCR rate in locally advanced READ [15, 16]. However, the specific reasons why irradiation plus immunotherapy showed better efficacy in READ remained unclear.

To sum up, we are still unable to identify who can benefit from CRT best in patients with READ. The molecular mechanisms of radiotherapy resistance in rectal cancer remain to be determined. Therefore, we comprehensively explored the differentially expressed genes (DEGs) between radiotherapy non-responder and responder of READ and the relationship between hub genes and tumor immune infiltration and the potential mechanisms, so as to screen out specific biomarkers to predict the sensitivity of radiotherapy and improve prognosis of READ patients treated with CRT.

## Methods

### Datasets and acquisition

The raw message RNA (mRNA) expression dataset of READ analyzed in this study was obtained from The Cancer Genome Atlas (TCGA) (https://portal.gdc.cancer.gov/) database, including 10 normal group and 167 tumor tissues. Another gene expression dataset was enrolled from the Gene Expression Omnibus (GEO) database (https://www.ncbi.nlm.nih.gov/geo/). The series Matrix File data file of GSE35452 from GEO was established on the annotation platform of GPL570. There were 46 cases in this dataset, including 24 preoperative radiotherapy READ responders and 22 preoperative radiotherapy non-responders. The samples were analyzed to screen DEGs. All of the data were available for free online.

### Functional enrichment analysis of the DEGs

DEGs sets were functionally annotated through the Metascape database (www.metascape.org) to explore the functional correlations of the gene sets comprehensively. Gene ontology (GO) analysis and Kyoto Encyclopedia of Genes and Genomes (KEGG) pathway analysis were performed on specific genes. Min overlap ≥ 3 & *P* ≤ 0.01 were considered statistically significant.

### Identification of hub genes by random survival forest analysis

Feature selection was performed using the randomForestSRC package. We used a random survival forest algorithm to rank the importance of prognostic related genes (nrep = 1000, which indicated that the number of iterations in the Monte Carlo simulation was 1000). We identified genes with relative importance > 0.3 as final marker genes.

### Analysis of immune cell infiltration on hub genes

The CIBERSORT algorithm was used to analyze the RNA-seq data of different subgroups of READ patients, to infer the relative proportions of 22 immune infiltrating cells, and to perform pearson correlation analysis on gene expression and immune cell content. *P* < 0.05 was considered statistically significant.

### Drug sensitivity analysis

Based on the largest pharmacogenomics database, named as Genomics of Drug Sensitivity in Cancer (GDSC, https://www.cancerrxgene.org/), we used the R package “pRRophetic” to predict the chemosensitivity of each tumor sample. Half maximal inhibitory concentration (IC50) estimating for each specific chemotherapeutic drug was assessed by regression method. Regression and prediction accuracy were tested with 10 cross-validation on the GDSC training set. Default values ​​were chosen for all parameters, including ‘combat’ to remove batch effects and to average replicate gene expression.

### Gene set variation analysis (GSVA)

GSVA ​​is a nonparametric and unsupervised method for evaluating enrichment of transcriptome gene set. By comprehensively scoring the gene set of interest, GSVA converts gene-level changes into pathway-level changes, and then judges the biological function of the sample. This study downloaded gene sets from the molecular signatures database, and the GSVA algorithm was used to comprehensively score each gene set, so as to evaluate the potential biological function changes of different samples.

### Gene set enrichment analysis (GSEA)

GSEA (http://www.broadinstitute.org/gsea) was used to identify genes that were differentially expressed between high and low expression groups based on expression profiles of READ patients. Gene sets were filtered using maximum and minimum gene set sizes of 500 and 15 genes, respectively. After 100 permutations, an enriched gene set was obtained based on a *P* < 0.05 and a false discovery rate (FDR) value of 0.25.

### Regulatory network analysis of hub genes

This study used the R package “RcisTarget” to predict transcription factors (TFs). All calculations performed by RcisTarget were based on motifs. The normalized enrichment score (NES) for motifs was depended on the total number of motifs in the database. In addition to the motifs annotated by the source data, we inferred further annotation files based on motif similarity and gene sequence. The first step in estimating the overexpression of each motif on a gene set was to calculate the area under the curve (AUC) for each motif-motif set pair. This was calculated from the recovery curves of the gene sets for motif ordering. The NES for each motif was calculated based on the AUC distribution of all motifs in the gene set. We used rcistarget.hg19.motifdb.cisbpont.500 bp for the Gene-motif rankings database.

### Genome-wide association study (GWAS analysis)

The Gene Atlas database (http://geneatlas.roslin.ed.ac.uk/) is a large database that documents associations between hundreds of traits and millions of variants using the UK Biobank cohort. These associations were calculated using 452,264 UK individuals in the UK Biobank database, covering a total of 778 phenotypes and 30 million loci.

### Validation of the protein expression levels of the hub genes via the human protein atlas

To further verify the protein expression levels of PLAGL2, ZNF337 and ALG10 in colorectal cancer and normal tissues, immunohistochemistry (IHC) data was downloaded from the Human Protein Atlas (HPA, http://www.proteinatlas.org). The HPA could provide IHC results of multiple proteins based on proteomics in both cancer tissues and normal tissues.

### Statistical analysis

All statistical analyses were performed using R language (version 3.6). Cox regression analysis was used to screen out prognostic genes. Survival analysis was performed by Kaplan-Meier method. Comparison between groups was performed by Wilcox test. *P* < 0.05 was considered statistically significant.

## Results

### Identification of DEGs of radio-sensitive samples in the READ cohort and functional enrichment of DEGs

**Fig. 1:**
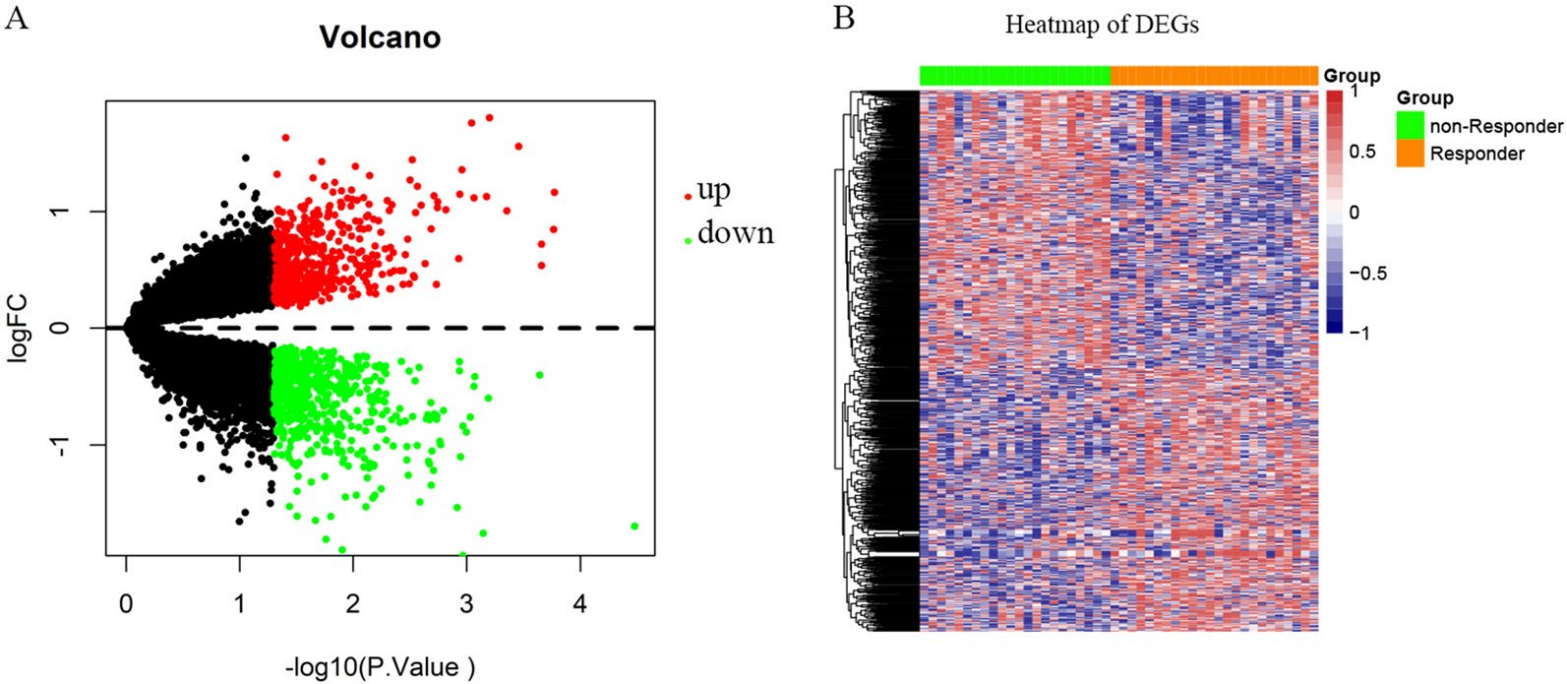
Volcano plot and heatmap of differentially expressed genes (DEGs). **A** The volcano plot of DEGs. Red: upregulated genes; Green: downregulated genes; **B** The heatmap of DEGs between non-responder (green) and responder (orange) of READ to radiotherapy. Blue: low expression level; Red: high expression level

We performed DEGs analysis on the GEO dataset GSE35452 by limma, and the results showed that 1,119 DEGs were differentially expressed between the radiotherapy responder and non-responder of READ based on the criteria of *P* < 0.05. Among that, 544 genes were up-regulated and 575 genes were down-regulated. The volcano plot and heatmap of DEGs were displayed in Fig. 1A and B. All of the upregulated and downregulated genes were demonstrated in Additional file 1: Table S1. To further investigate the function and pathways of the DEGs, metascape enrichment analysis was used and the results showed that these candidate genes were mainly enriched in pathways such as chemical synaptic transmission, regulation of hormone levels, and olefinic compound metabolic process and so on (See Additional file 1:  Fig. S1). At the same time, we performed protein-protein interaction (PPI) network analysis of DEGs by Cytoscape software. The results demonstrated that the network connections of DEGs were close and complex, which was shown in Additional file 1:  Fig. S2.

### Random survival forest analysis of DEGs and identification of three hub genes of PLAGL2, ZNF337 and ALG10

**Fig. 2:**
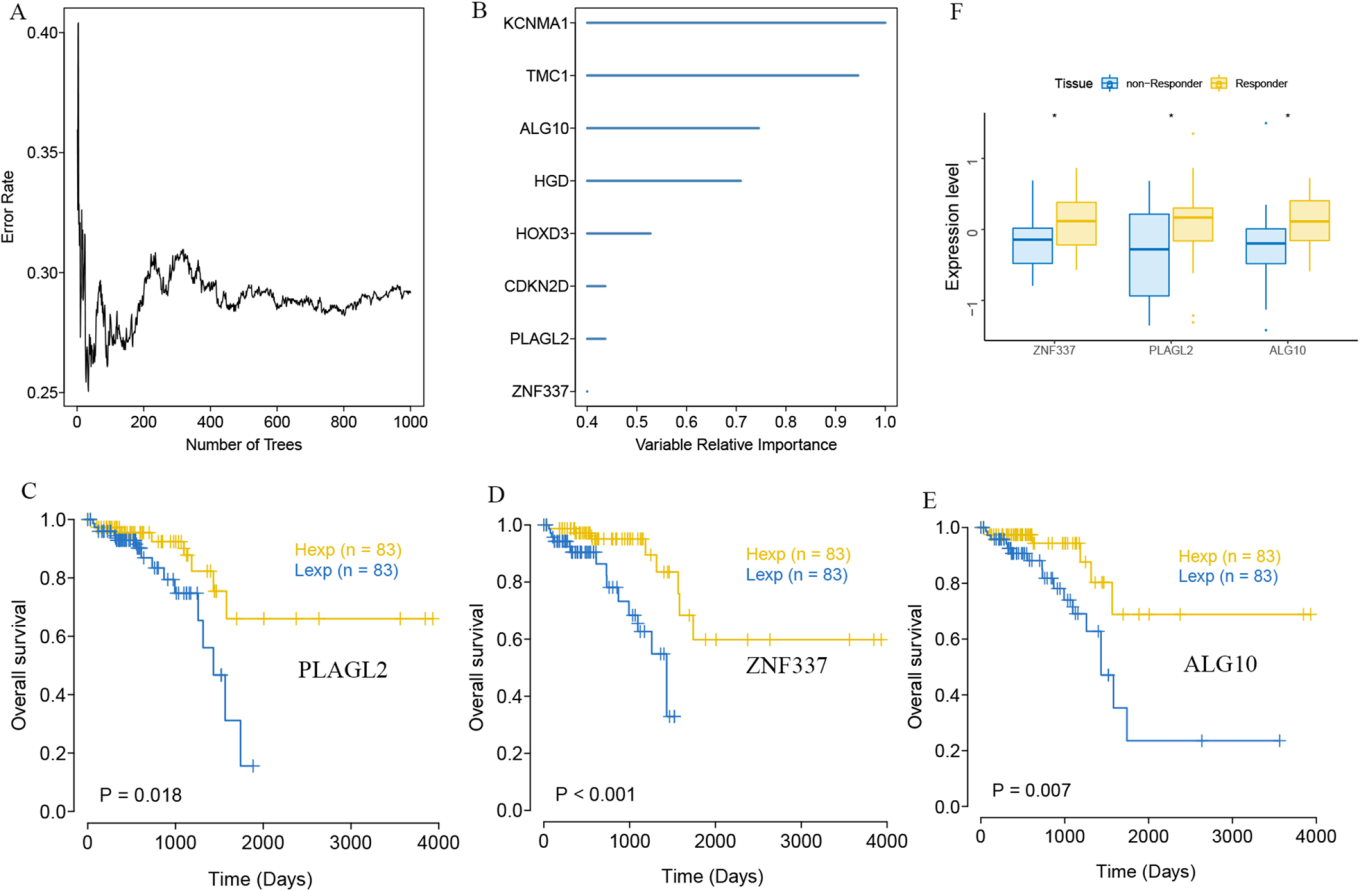
Random survival forest analysis of DEGs in TCGA-READ cohort. **A** Random survival forest analysis of DEGs. **B** Eight genes were identified as finial markers with variable relative importance > 0.3. **C** High expression of *PLAGL2*, *ZNF337* **D** and *ALG10* **E** were significantly associated with better overall survival compared to low expression by Kaplan-Meier survival analysis (*P* = 0.018, *P* < 0.001 and *P* = 0.007, respectively). **F**. The expression of *ZNF337*, *PLAGL2* and *ALG10* was significant higher in radiotherapy responder READ patients than non-responder patients (* represented *P* < 0.05)

In order to further find out the core genes that affected rectal cancer among the DEGs, we selected the DEGs in the TCGA-READ cohort for random survival forest analysis. Genes with relative importance > 0.3 were identified as final markers. Finally, eight genes were consistent with our screening threshold, including *KCNMA1*, *TMC1*, *ALG10*, *HGD*, *HOXD3*, *CDKN2D*, *PLAGL2* and *ZNF337*, shown as Fig. 2A and B. Among these eight genes, only three hub genes had statistical significance by Kaplan-Meier survival analysis, which were *PLAGL2*, *ZNF337* and *ALG10* (Fig. 2C, D and E). The results showed that high expression of *PLAGL2*, *ZNF337* and *ALG10* were significantly associated with better overall survival compared to low expression (*P* = 0.018, *P* < 0.001 and *P* = 0.007, respectively). Moreover, the expression of the three genes was significant higher in radiotherapy responder READ patients than in non-responder patients (Fig. 2F).

### Exploration of the clinical predictive value of three hub genes based on multi-omics studies

**Fig. 3:**
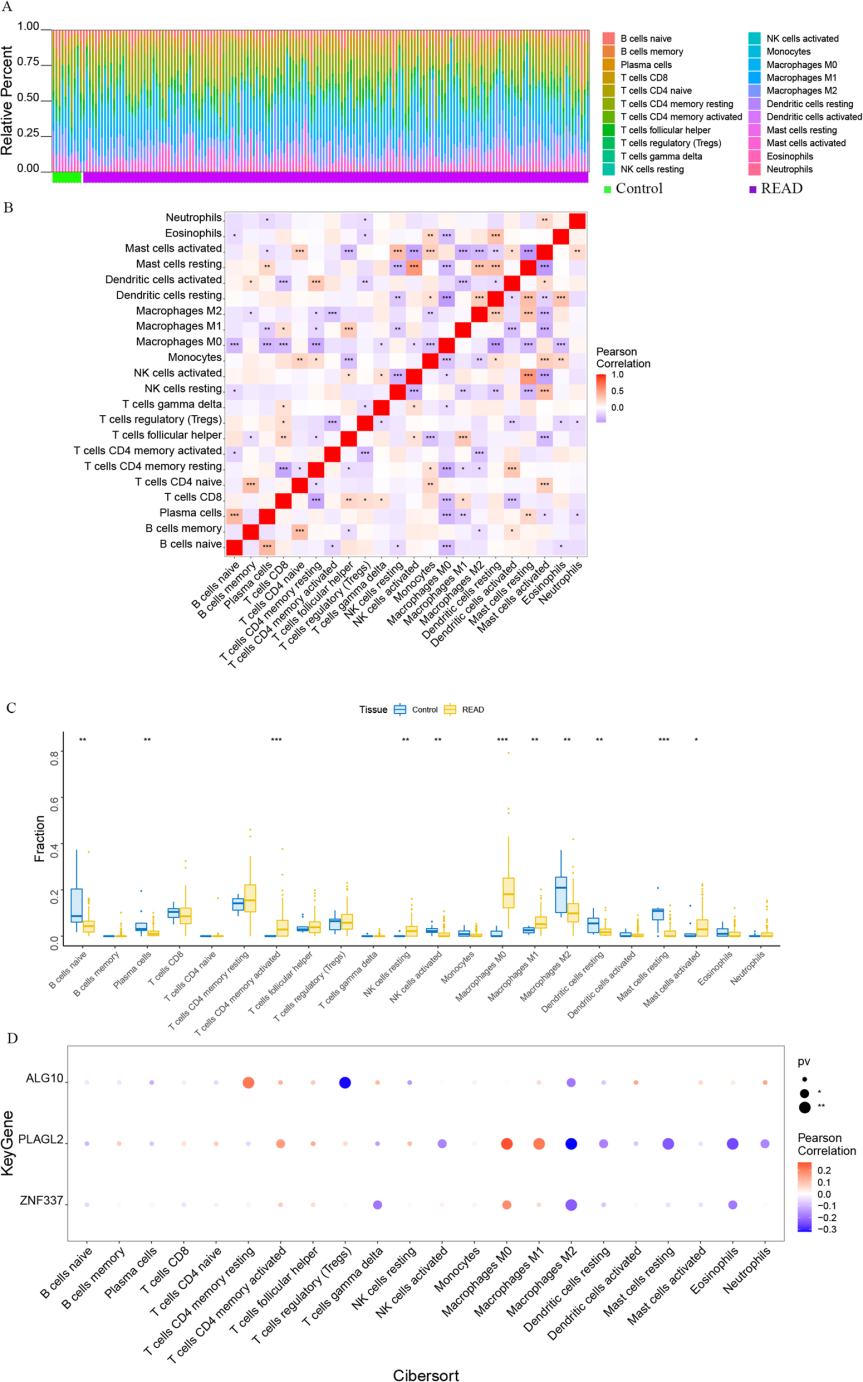
Composition of immune infiltrating cells in association with three hub genes in the cohort retrieved from TCGA. **A**: Percentage of immune cells between the normal group and the READ patients. Green: the normal control group; Purple: the READ group. **B**: Interaction analysis among 22 different immune cells in READ patients (* represented *P* < 0.05, ** represented *P* < 0.01 and *** represented *P* < 0.001). **C**. Comparisons of immune cells between in the normal control group and READ tissue group (* represented *P* < 0.05, ** represented *P* < 0.01 and *** represented *P* < 0.001 between the two group). **D**. Bubble map for the correlations between three hub genes (*ALG 10*, *PLAGL2* and *ZNF337*) and tumor immune infiltration cells (* represented *P* < 0.05, ** represented *P* < 0.01; The bigger the circle, the closer the *P* - value was to zero; The redder the color, the stronger the positive correlation; The deeper of the purple color, the stronger the negative correlation)

The tumor microenvironment is mainly composed of tumor-associated fibroblasts, immune cells, extracellular matrix, various growth factors, inflammatory factors, special physicochemical characteristics, and cancer cells themselves. Tumor microenvironment significantly affects the diagnosis, survival and treatment sensitivity of malignant tumors. By analyzing the relationship between the expression of hub genes and tumor immune infiltration, the potential molecular mechanism of the hub genes that affecting the progression of rectal cancer was further explored. The proportion of immune infiltrating cells in each patient and the pearson correlation between immune cells were shown in Fig. 3A and B. The proportion of T cells CD4 memory activated, NK cells resting and macrophages M0 were significantly higher in READ patients than in the normal patients (Fig. 3C). *PLAGL2* was significantly positively correlated with macrophages M0, macrophages M1, etc., and significantly negatively correlated with macrophages M2, dendritic cells resting, etc.; *ZNF337* was significantly positively correlated with macrophages M0, etc., and significantly negatively correlated with macrophages M2, etc.; Moreover, *ALG10* was significantly positively correlated with T cells CD4 memory resting and significantly negatively correlated with T cells regulatory (Tregs) (Fig. 3D).

Furthermore, we obtained the correlations between the three hub genes and different immune-related genes from the TISIDB database, including chemokines-related, immunoinhibitor-related, MHC-related, immunostimulatory-related and receptor-related genes. The results showed that *ALG10*, *ZNF337* and *PLAGL2* were positively or negatively correlated with multiple immune-related genes and the detail was displayed in Additional file 1:  Fig. S3.

It was explicit that fluorouracil-based chemoradiotherapy was the standard treatment of the locally advanced READ. We further studied the sensitivity of chemotherapeutic drugs between different expression levels of hub genes based on GDSC database using the R package “pRRophetic”. The results showed that the expression of *ZNF337* and *ALG10* could affect the sensitivity of paclitaxel, metformin, bryostatin.1, dasatinib, gefitinib and imatinib. However, *PLAGL2* had no effect on the IC50 of dasatinib and imatinib. Regrettably, the data of fluorouracil-base chemotherapeutic agents were absent in the GDSC database (See Additional file 1: Fig. S4).

### Study of the relationship between three hub genes and disease-related genes

**Fig. 4:**
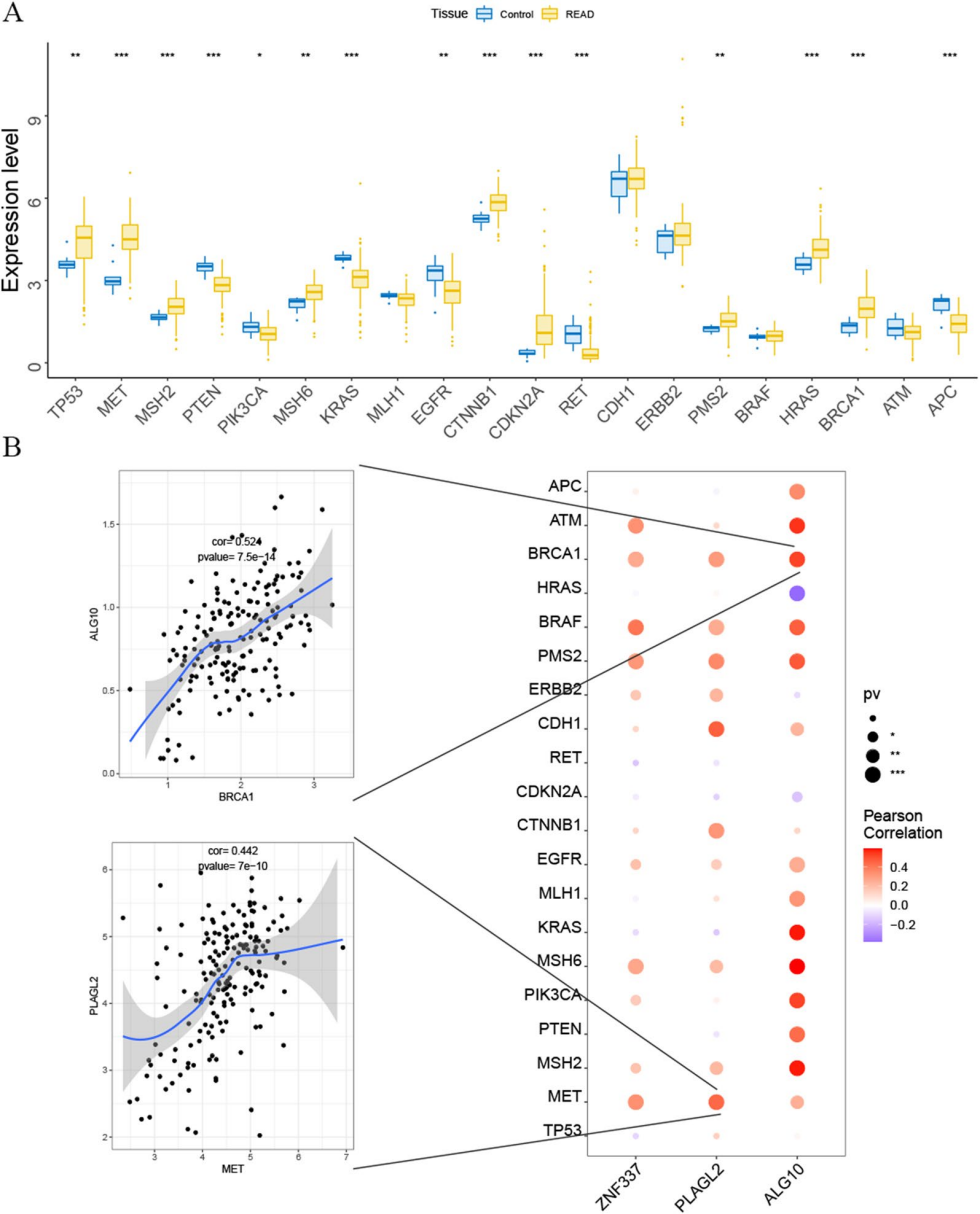
The relationship of hub genes and the disease-related genes (* represented *P* < 0.05, ** represented *P* < 0.01 and *** represented *P* < 0.001). **A** The comparisons of the expression of multiple disease-related genes between the control and READ patients. **B** Bubble map for the pearson correlations between three hub genes (*ALG 10*, *PLAGL2* and *ZNF337*) and disease-related genes. (The bigger the circle, the closer the *P* - value was to zero; The redder the color, the stronger the positive correlation; The deeper of the purple color, the stronger the negative correlation)

The disease genes related with the tumorigenesis of READ ware obtained through the GeneCards database (https://www.genecards.org/). The results showed the expressions of multiple disease-related genes were significant different between control group and READ group, which included genes of *TP53*, *MET*, *MSH2*, *PTEN*, *PIK3CA*, *MSH6*, *KRAS*, *EGFR*, *CTNNB1*, *CDKN2A*, *RET*, *PMS2*, *HRAS*, *BRCA1* and *APC* (Fig. 4A). The pearson correlation analysis pointed out that hub genes of *ZNF337*, *PLAGL2* and *ALG10* were significantly associated with the expression of various disease-related genes. As shown in Fig. 4B, high expression of *ALG10* was positively related to the expression of *BRCA1*, *APC*, *KRAS*, and etc.; high expression of *PLAGL2* correlated with higher expression of *MET*, *MSH2*, *CDH1*, and etc.; *ZNF337* had a positive relationship with *MET*, *MSH6*, *BRAF* and so on.

### Discussion on specific signaling mechanisms related to hub genes of PLAGL2, ZNF337 and ALG10

**Fig. 5:**
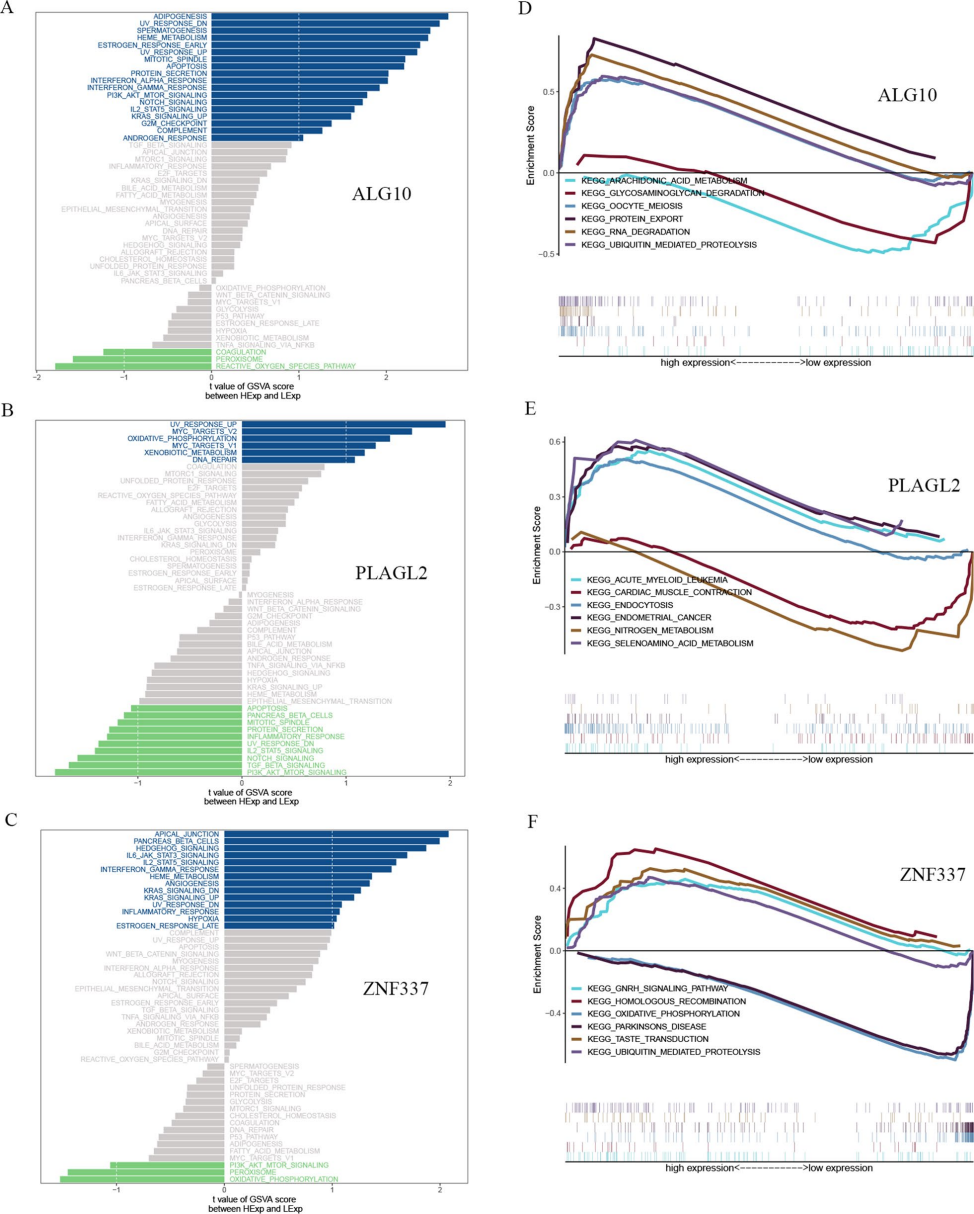
GSVA and GSEA analysis of high and low expression of *ALG10*, *PLAGL2* and *ZNF337*. **A** GSVA of *ALG10*; **B**: GSVA of *PLAGL2*; **C**: GSVA of *ZNF337*; **D**: GSEA of *ALG10*; **E**: GSEA of *PLAGL2*; **F**: GSEA of *ZNF337*.

We next analyzed the specific signaling pathways involved in the three hub genes, and explored the effect of candidate genes on the signaling pathways related to disease progression. GSVA results showed that high expression of *ALG10* mainly enriched adipogenesis, UV-response-down, apoptosis, *PI3K*-*AKT*-*mTOR* signaling, *NOTCH*, G2M checkpoint and other signal pathways. High expression of *PLAGL2* mainly enriched signaling pathways such as UV response-up, MYC targets V2, oxidative phosphorylation and DNA-repair. Low expression of *PLAGL2* mainly enriched in apoptosis, *NOTCH* signaling, *TGFβ* signaling and *PI3K*-*AKT*-*mTOR* signaling pathways. High expression of *ZNF337* mainly enriched signaling pathways such as apical junction, *IL6*-*JAK*-*STAT3* signaling, *IL2*-*STAT5* signaling, angiogenesis pathway and others (Fig. 5A, B and C). In addition, we also performed GSEA analysis on these genes, and the enriched pathways of hub genes were shown in the Fig. 5D and E F. The results showed that high expression of *ALG10* enriched in protein export, RNA degradation, ubiquitin mediated proteolysis and so on. High expression of *PLAGL2* enriched in signaling pathways such as endocytosis, endometrial cancer and others. Low expression of *PLAGL2* enriched in nitrogen metabolism . High expression of *ZNF337* enriched in pathways like homologous recombination, *GHRN* signaling and others.

### Construction of nomogram and development of calibration curves to predict the outcome of patients with READ

**Fig. 6:**
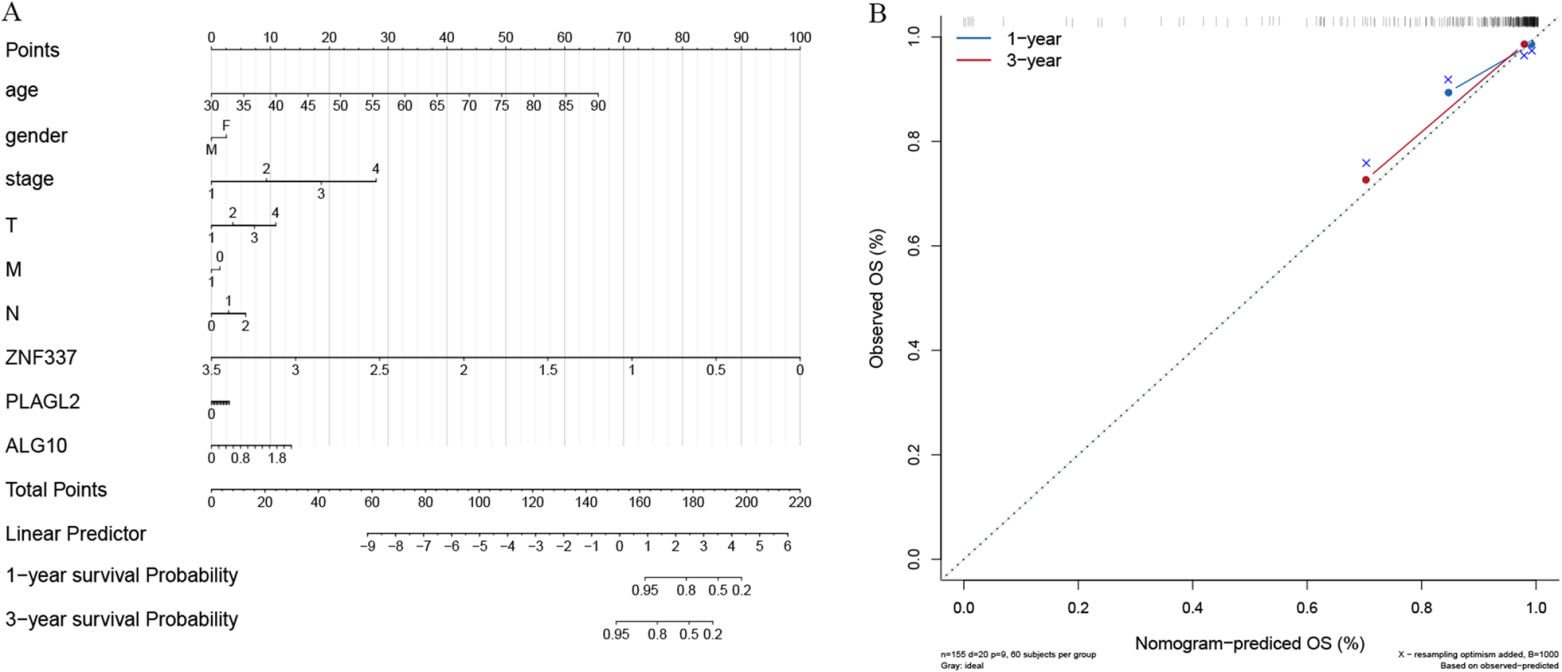
Nomogram for prediction of the outcome of patients with READ. **A**: Nomogram was constructed based on the expression of *ALG10*, *PLAGL2* and *ZNF337* and the clinical parameters. **B**: Calibration curves of nomogram for predicting OS at 1-year and 3-year in the TCGA READ dataset

A nomogram was constructed using the TCGA READ dataset based on the expression of *ALG10*, *PLAGL2* and *ZNF337* and the clinical characteristics including age, gender, stage, tumor (T), lymph node (N) and metastasis (M) stage. The logistic regression analysis showed that the clinical parameters and three hub genes of *ALG10*, *PLAGL2* and *ZNF337* had different degrees of contributions in the scoring process of READ at different stages. By scoring the features mentioned above, the higher the total points, the poorer the 1-year and 3-year survival probability (Fig. 6A). At the same time, the calibration curves for the probabilities of 1-year and 3-year OS revealed that the nomogram-predicted OS was in good agreement with the observed OS (Fig. 6B).

### Regulatory network analysis of hub genes and competitive endogenous RNA (ceRNA) network analysis of PLAGL2

**Fig. 7:**
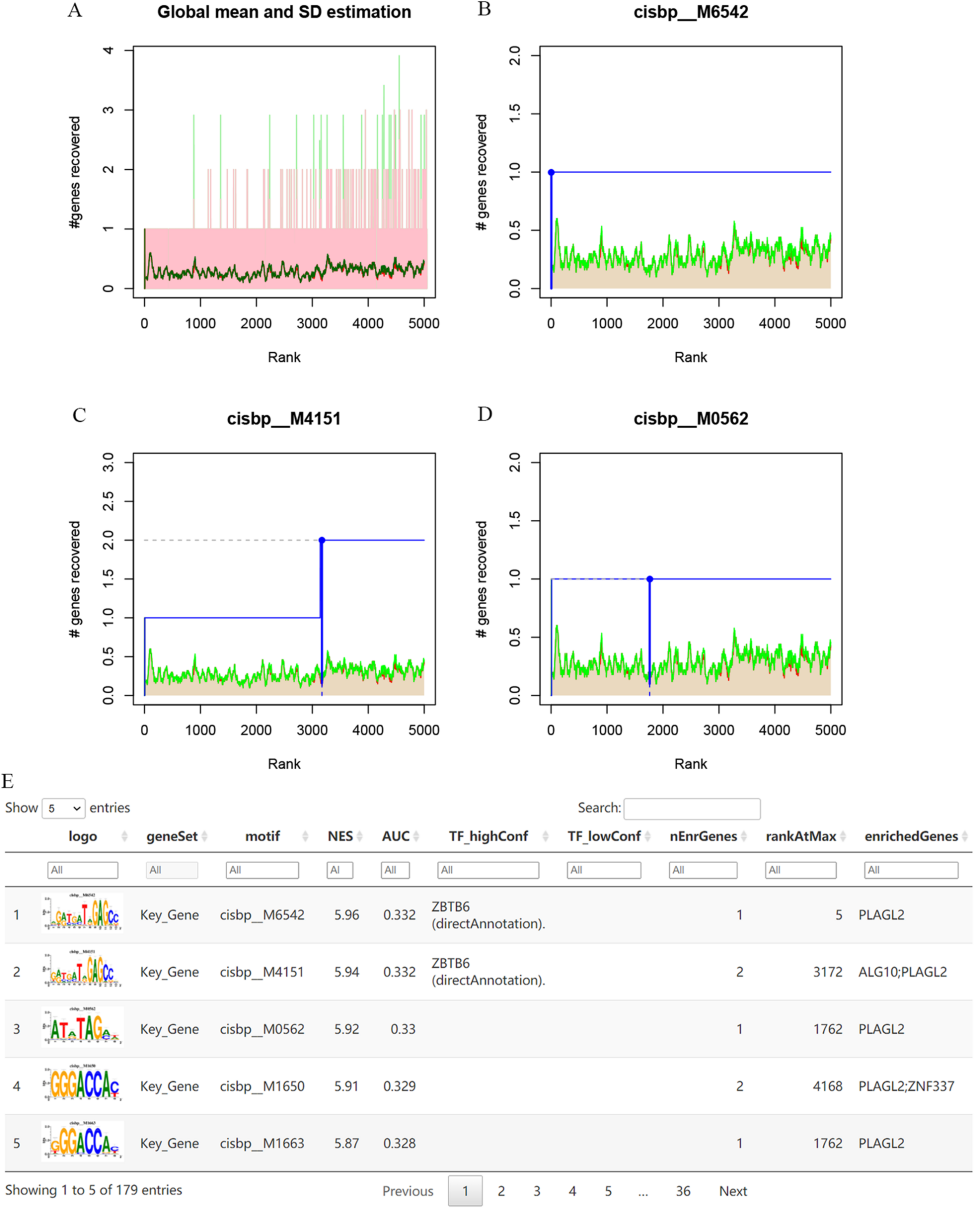
Regulatory network analysis of hub genes. **A**: the enrichment analysis of transcription factors of *ALG10*, *PLAGL2* and *ZNF337* by “RcisTarget” from R package; **B**: the motif of cisbp_M6542; **C**: the motif of cisbp_M4151; **D**: the motif of cisbp_M0562; **E**: part enriched motifs and corresponding TFs for hub genes

In the study, three hub genes of *ALG10*, *PLAGL2* and *ZNF337* were evaluated in the process of predicting relevant TFs. The analysis demonstrated that they were regulated by multiple TFs. Therefore, motif enrichment analysis was performed for these TFs (Fig. 7A). The results showed that the motif with the highest NES was annotated as cisbp_M6542 (NES was 5.96) (Fig. 7B), followed by cisbp_M4151 with NES of 5.94 and cisbp_M0562 with NES of 5.92 (Fig. 7C and D). The gene enriched in motif of cisbp_M6542 was *PLAGL2* and the predicted upstream TF was *ZBTB6*. A regulatory network of TF (*ZBTB6*)-mRNA (*PLAGL2*) was established. We displayed a fraction of enriched motifs and corresponding TFs for hub genes in Fig. 7E.

**Fig. 8:**
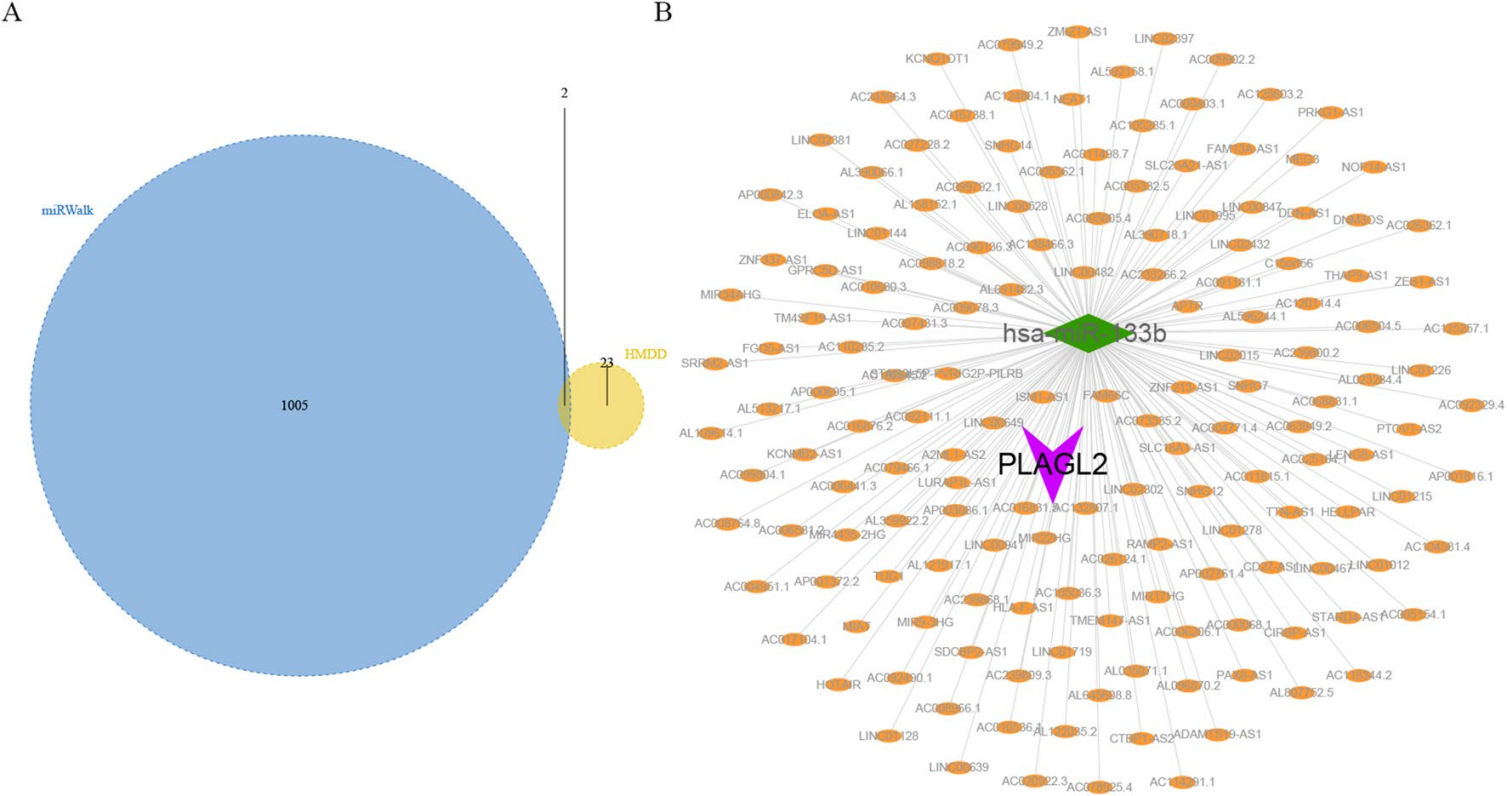
CeRNA network analysis of hub genes. **A**: Twenty-five miRNAs related to READ and 1,007 miRNAs related to *ALG10*, *PLAGL2* and *ZNF337* were identified from HMDD and miRWalk databases respectively; **B**: A ceRNA network of mRNA (*PLAGL2*)-miRNA (has-miR-133b)-lncRNA (157 in total) was constructed

We obtained 25 READ-related microRNAs (miRNAs) from the Human MicroRNA Disease Database (HMDD) (http://www.cuilab.cn/hmdd). Possible miRNAs and long non-coding RNAs (lncRNAs) for *ALG10*, *PLAGL2* and *ZNF337* were predicted through miRWalk database and ENCORI database, respectively. First, the mRNA-miRNA relationship pairs related to mRNAs of the three hub genes were extracted from the miRWalk database and a total of 1,007 miRNAs were obtained. Only mRNA-miRNA relationship pairs that were disease-related miRNAs were retained. Finally, only two mRNAs and two miRNAs were included (Fig. 8A). Second, the interacting lncRNAs based on ENCORI database were predicted according to the two miRNAs. Finally, a total of 157 pairs of interactions were predicted, which included 1 miRNA and 157 lncRNAs. The results showed *PLAGL2* was regulated by has-miR-133b and the a complex ceRNA network of miRNA (has-miR-133b)-lncRNA was constructed by cytoscape software (Fig. 8B).

### GWAS analysis of hub genes

**Fig. 9:**
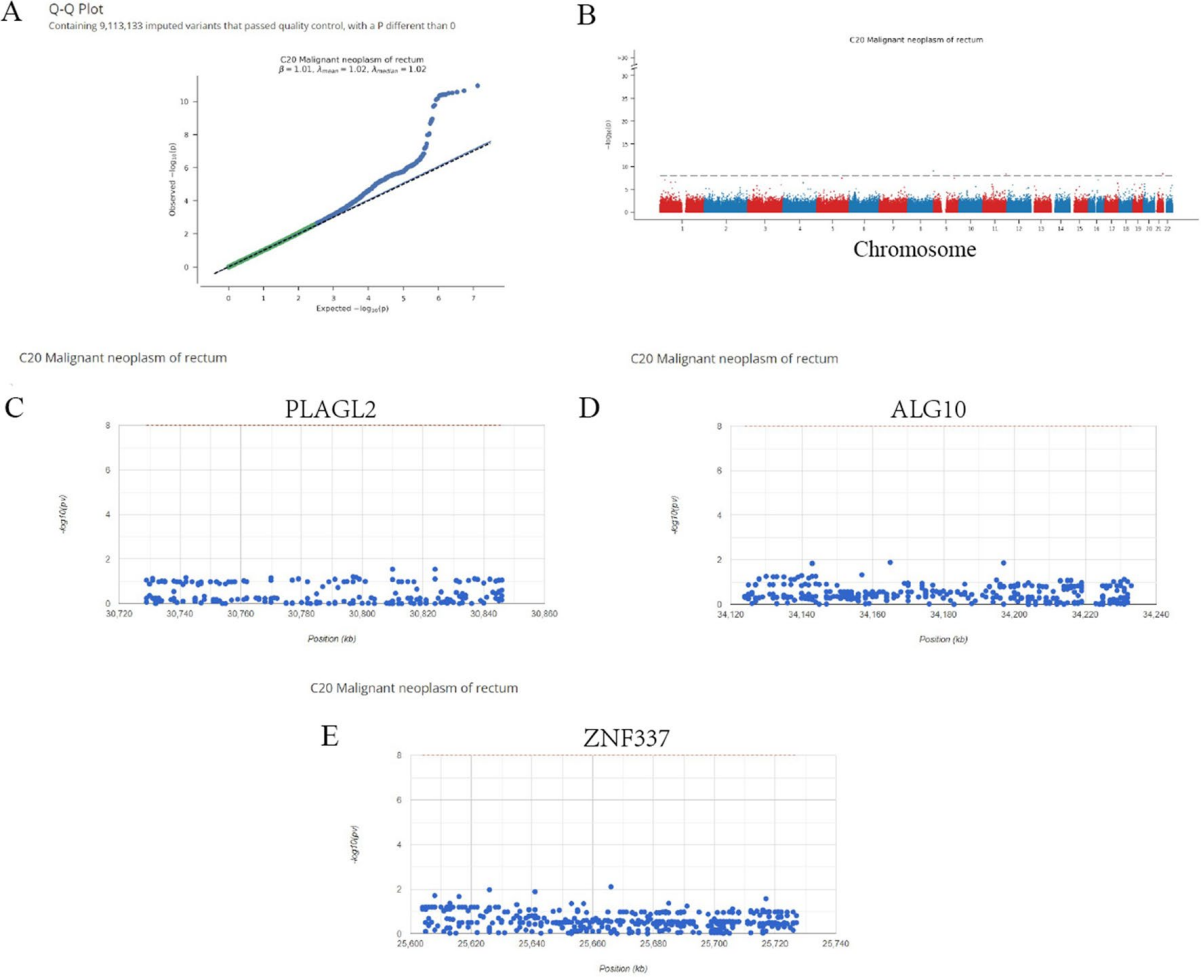
Results of the GWAS analysis. **A**: Q - Q plot of the GWAS; **B**: Manhattan plot of the GWAS; **C**: *PLAGL2* was located in the pathogenic region of chromosome 20. **D**: *ALG10* was located in the pathogenic region of chromosome 12. **E**: *ZNF337* was located in the pathogenic region of chromosome 20

Next, the pathogenic regions of 3 hub genes in READ were identified by analyzing the GWAS data (Fig. 9A and B). The single nucleotide polymorphism (SNP) pathogenic regions corresponding to hub genes of *PLAGL2*, *ZNF337* and *ALG10* were also displayed. The results showed *PLAGL2* and *ZNF337* were located in the pathogenic region of chromosome 20 and *ALG10* was located in the pathogenic region of chromosome 12 (Fig. 9C, D and E).

### Validation of the expression levels of PLAGL2, ZNF337 and ALG10 in clinical samples

**Fig. 10:**
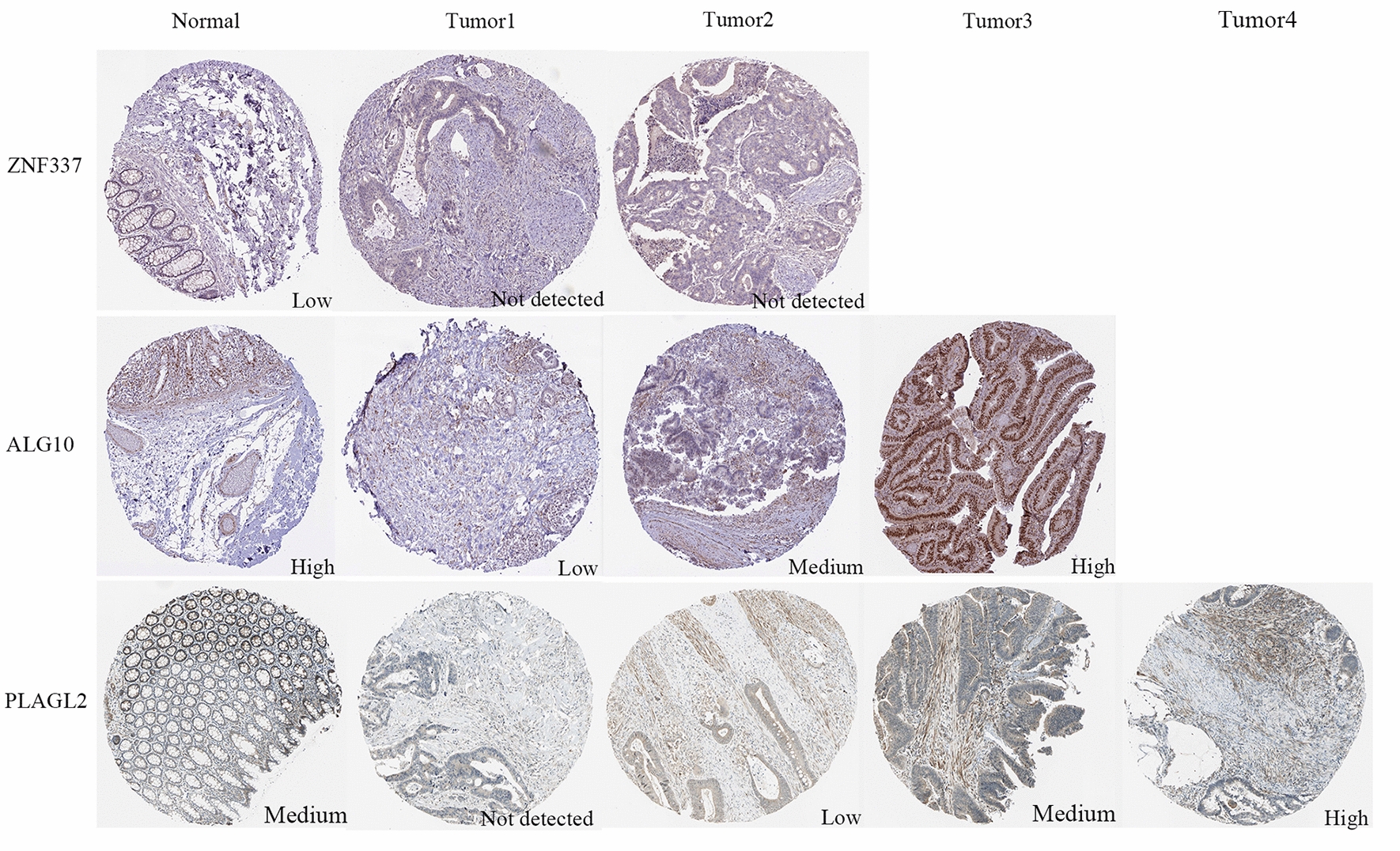
The protein expression levels of ZNF337, ALG10 and PLAGL2 in rectum tissues and colorectal cancers from HPA online database

IHC results of the protein expression of ZNF337, ALG10 and PLAGL2 from HPA database were displayed in Fig. 10. ZNF337 was expressed at a low level in rectum normal tissues. There was no information of the expression of ZNF337 in READ on the HPA database, so we identified the expression of ZNF337 in colon cancer instead. The protein expression level of ZNF337 was not detected in colon cancers. ALG10 was expressed at a high level in rectum normal tissues and had various expression levels in READ patients, which was from low and medium to high expression. Moreover, PLAGL2 was expressed at a medium level in rectum normal tissues and also had diverse expression levels in READ patients, which was from not detected and low expression to medium and high expression. The different expression levels of hub genes might explain the inherent biological characteristics differences and individual difference in radiosensitivity in READ patients.

## Discussion

A comprehensive understanding about the mechanism of radiotherapy sensitivity is the key to improve the outcomes of READ. Till now, a comprehensive research of TF-miRNA-target genes regulatory network of radio-resistance based on clinical specimens in READ is still absent. Hence, we explored the biomarkers and potential mechanism of radio-resistance in READ patients by bioinformatics analyses systematically. The flowchart of our study was displayed in Additional file 1: Fig. S5.

In this study, the DEGs between radiotherapy responder and non-responder of READ patients were identified based on GEO dataset GSE35452 by limma. Totally, 544 up-regulated and 575 down-regulated were screened out. Functional enrichment analysis showed DEGs significantly enriched in multiple cancer biological processes and the interrelationships between DEGs were complex by PPI analysis. Random survival forest analysis of DEGs from the TCGA-READ cohort demonstrated eight genes accorded with the threshold, including *KCNMA1*, *TMC1*, *ALG10*, *HGD*, *HOXD3*, *CDKN2D*, *PLAGL2* and *ZNF337*. Finally, *PLAGL2*, *ZNF337* and *ALG10* were significantly associated with the survival of READ patients and identified as hub genes of current study. Analysis based on exploration of the clinical predictive value showed *PLAGL2*, *ZNF337* and *ALG10* were significantly associated with tumor immune infiltration, different immune-related genes and sensitivity of chemotherapeutic drugs. Moreover, these three hub genes were correlated with the expression of various disease-related genes, including *BRCA1*, *APC*, *KRAS*, *MET*, *MSH2*, *CDH1*, *MSH6*, *BRAF* and so on. GSVA and GSEA analysis indicated different expression levels of *PLAGL2*, *ZNF337* and *ALG10* might influence various signaling pathways related to disease progression, such as *PLAGL2* affected DNA repair, oxidative phosphorylation, apoptosis, *NOTCH* signaling, *TGFβ* signaling and *PI3K*-*AKT*-*mTOR* signaling pathways. A nomogram was constructed to predict the outcomes of READ according to different expression of *ALG10*, *PLAGL2* and *ZNF337* and the clinical characteristics. The calibration curves showed the nomogram-predicted OS had a high prediction in READ patients.

Finally, regulatory network showed that *PLAGL2*, *ZNF337* and *ALG10* were regulated by various TFs and a regulatory network of TF (*ZBTB6*)-mRNA (*PLAGL2*) was identified. Moreover, a ceRNA network of miRNA (has-miR-133b)-lncRNA which regulated *PLAGL2* target gene was constructed. GWAS analysis displayed the pathogenic regions of *PLAGL2*, *ZNF337* was located in chromosome 20 and A*LG10* was in chromosome 12. In addition, we further validated the expression of the three genes in clinical samples based on HPA online database and the results showed the proteins expression levels varied widely in READ patients, which might indicate and reflect the internal radiosensitivity difference between diverse cancer patients.

*PLAGL2*, named as pleiomorphic adenoma gene-like 2, and also known as *PLAG1* like zinc finger 2 and *ZNF900*, is a zinc-finer protein that recognizes DNA and/or RNA and associated with the tumorigenesis of several malignancies [17]. It had been shown that overexpression of *PLAGL2* was involved in the process of carcinogenicity of ovarian cancer cells through modulation of lncRNA ARAP1-AS1/miR-4735-3p/PLAGL2 axis [18]. Another research indicated *PLAGL2* contributed to the development of lung adenocarcinoma in mice model [19]. Also, highly-expressed *PLAGL2* could impede differentiation and expedite self-renewal capacity by modulating Wnt/β-catenin signaling pathway in neural stem cells and gliomas [20]. Moreover, *PLAGL2* had a critical biological role in promoting the malignant phenotypes of gastric cancer cells through USP37-mediated deubiquitination of Snail protein [21]. Other study revealed that *PLAGL2* expression was also associated with an intestinal epithelial stem cell signature through enhancing the expression of a transcriptional regulator of *ASCL2* and activating Wnt gene expression in intestinal epithelial cells [22]. Besides, *PLAGL2* might promote epithelial-mesenchymal transition and increase metastasis via β-catenin-dependent regulation of *ZEB1* in colorectal cancer [23]. It was also reported that *PLAGL2* was associated with chemotherapeutic drug resistance of adriamycin in breast cancer by activating Wnt pathway as well [24]. To our knowledge, the biological characteristics between colon cancer and rectal cancer differed obviously and no study investigated the role of *PLAGL2* in the process of radio-resistance in rectal cancer.

*ALG10*, fully defined as asparagine-linked alpha-1,2-glucosyltransferase, and is also known as *DIE2* or *KCR1* or *ALG10A*, encodes a membrane-associated protein that participates in putting the third glucose residue to the lipid-linked oligosaccharide precursor for N-linked glycosylation [25]. An article published recently concluded that high expression of *ALG10* facilitated the glycosylation of *TGFBR2*, stimulated *TGF-β* pathway and thus promoted the stemness of colorectal cancer cells [26]. However, the role of *ALG10* in chemoradiotherapy resistance of rectal cancer had not been rigorously studied and discussed. *ZNF337*, named as zinc finger protein 337, encodes a zinc finger domain containing protein and its biological function has yet to be determined and the role in tumorigenesis and cancer progression are still unclear.

Evidence showed colorectal cancer cells had extensive mutational diversification and exhibited higher somatic mutation burdens than normal colorectal stem cells and individual cancer cells experienced inherited differences. Moreover, the responses to anticancer drugs were markedly different between even closely relevant cells in the same tumor [27]. In our study, the HPA database showed the expression of the hub genes differed in individual colorectal cancer, which was consistent with the above findings. In addition, some clinical trials are currently investigating the efficacy of the combination of radiotherapy and immunotherapy as neoadjuvant treatment before operation in locally advanced READ. Since the preliminary results have showed the tumor regression rates were apparently better in radiotherapy and immunotherapy combination group than the standard CRT strategy, where the pCR rate could reach to 48% in all cohort and 60% in microsatellite instability-high cohort [15, 16]. However, the underlying synergetic mechanism was still uncleared. In this study, we found hub genes that related to the response of radiotherapy had significant correlations with immune infiltrating cells and immune-related genes, which might reveal the potential mechanism of radioimmunotherapy combination.

It had been demonstrated that miRNA and lncRNA took critical roles in the process of gene regulation and cancer biology [28]. MiRNAs, known as small non-coding RNAs, could bind with mRNA and control its expression. The ceRNA networks, where lncRNA acted as miRNA to modulate gene expression, participated in cancer development and progression [29]. In order to comprehensively recognize the role and potential mechanism of the three hub gens on radiosensitivity and prognosis of READ, the miRNA-related regulatory network and ceRNA network were constructed in current study.

However, there are still some limitations and shortages in our research. First, the sample sizes retrieved from TCGA and GEO databases were small. Second, the results were lack of experimental validations in vivo and in vitro. Despite these shortcomings, the preliminary study can still provide very meaningful and constructive findings. In the subsequent studies, we will further confirm the role of the hub genes of *PLAGL2*, *ZNF337* and *ALG10* in radio-resistance of READ in series experiments. The effect of the overexpression and knockdown of *PLAGL2*, *ZNF337* and *ALG10* on cell cycle distribution, proliferation, colony-forming, apoptosis and invasiveness after irradiation will be investigated in human rectal cell lines and in vivo. Besides, the most relevant signaling pathways and TF (*ZBTB6*)-miRNA (has-miR-133b)-mRNA (*PLAGL2*) regulatory network will also be explored integrally.

## Conclusion

Taken together, the DEGs and three hub genes of *PLAGL2*, *ZNF337* and *ALG10* were identified in radiotherapy responders in READ. The relationship between hub genes and tumor immune infiltration, immune-related genes, sensitivity of chemotherapeutics, disease-related genes of READ, enriched signaling pathways, TF-miRNA-mRNA and ceRNA regulatory networks were systematically illustrated. Otherwise, the prognostic nomogram containing clinicopathological features and hub genes was constructed and calibration curves was established, which could excellently predict the survival of READ. Hence, the preliminary but relatively comprehensive study provided a new perspective for the understanding of radiosensitivity in READ, and predicted potential biomarkers and molecular mechanism for the radiotherapy and prognosis of READ. Future articles in this series will go into more details about the effects of hub genes of *PLAGL2*, *ZNF337* and *ALG10* on the phenotype of rectal cancer cells after irradiation in vitro and in vivo. The potential signaling pathway and molecular mechanisms of hub genes related to radiotherapy sensitivity will also be investigated in depth.

## Supplementary Materials

**Additional file 1: Fig. S1. **Functional enrichmentof DEGs by metascape enrichment analysis. **Fig. S2. **Protein-protein interaction network analysis of DEGs by Cytoscape analysis. Circles represented genes, and lines represented the interaction of proteins between genes. **Fig. S3. **The correlations between the expression of hub genes (*ALG 10*, *PLAGL2* and *ZNF337*) and different immune-related genes from the TISIDB database. A. Correlated with chemokines-related genes; B. Correlated with immunoinhibitor-related genes; C. Correlated with MHC-related genes; D. Correlated with immunostimulatory-related genes; E: Correlated with receptor-related genes. (* represented *P *< 0.05, ** represented *P *< 0.01 and *** represented *P *< 0.001; The redder the color, the stronger the positive correlation; The deeper the purple color, the stronger the negative correlation). **Fig. S4. **The sensitivity analysis of chemotherapeuticdrugs between different expression levels of hub genes based on GDSC database. A: Bryostatin.1; B: Dasatinib; C: Gefitinib; D: Imatinib; E: Metformin; F: Paclitaxel. **Fig. S5. **The flowchart of the current study. **Table S1. **Information of the DEGs identified from database. A total of 1119 DEGs were identified from GSE35452 dataset, including 544 up-regulated genes and 575 down-regulated genes in READ cohort.

## Data Availability

The datasets used and/or analyzed during the current study are available from the corresponding author on reasonable request. The data that support the results of current study is available on The Cancer Genome Atlas (TCGA) and Gene Expression Omnibus (GEO) websites.

## Abbreviation

READ: Rectum adenocarcinoma
CRT: Chemoradiotherapy
TNT: Total neoadjuvant chemotherapy
PCR: Pathological complete response
DFS: Disease-free survival
OS: Overall survival
TNM: Tumor-node-metastasis
DSB: Double-strand breaks
DEGs: Differentially expressed genes
mRNA: Message RNA
TCGA: The Cancer Genome Atlas
GEO: Gene Expression Omnibus
GO: Gene ontology
KEGG: Kyoto Encyclopedia of Genes and Genomes
GDSC: Genomics of Drug Sensitivity in Cancer
GSVA: Gene set variation analysis
GSEA: Gene set enrichment analysis
TFs: Transcription factors
NES: Normalized enrichment score
AUC: Area under the curve
IHC: Immunohistochemistry
HPA: Human Protein Atlas
PPI: Protein-protein interaction
CeRNA: Competitive endogenous RNA
HMDD: Human MicroRNA Disease Database
lncRNAs: Long non-coding RNAs
SNP: Single nucleotide polymorphism
PLAGL2: Pleiomorphic adenoma gene-like 2
ALG10: Asparagine-linked alpha-1,2-glucosyltransferase
ZNF337: Zinc finger protein 337
