# Supplementary material for: Identification of hub genes and potential molecular mechanisms related to radiotherapy sensitivity in rectal cancer based on multiple datasets
Source: J Transl Med. 2023 Mar 6;21:176. doi: 10.1186/s12967-023-04029-2 (PMC9987056; doi:10.1186/s12967-023-04029-2)
Supplement: Supplementary file 1 — Additional file 1: Fig. S1. Functional enrichmentof DEGs by metascape enrichment analysis. Fig. S2. Protein-protein interaction network analysis of DEGs by Cytoscape analysis. Circles represented genes, and lines represented the interaction of proteins between genes. Fig. S3. The correlations between the expression of hub genes (ALG 10, PLAGL2 and ZNF337) and different immune-related genes from the TISIDB database. A. Correlated with chemokines-related genes; B. Correlated with immunoinhibitor-related genes; C. Correlated with MHC-related genes; D. Correlated with immunostimulatory-related genes; E: Correlated with receptor-related genes. (* represented P < 0.05, ** represented P < 0.01 and *** represented P < 0.001; The redder the color, the stronger the positive correlation; The deeper the purple color, the stronger the negative correlation). Fig. S4. The sensitivity analysis of chemotherapeuticdrugs between different expression levels of hub genes based on GDSC database. A: Bryostatin.1; B: Dasatinib; C: Gefitinib; D: Imatinib; E: Metformin; F: Paclitaxel. Fig. S5. The flowchart of the current study. Table S1. Information of the DEGs identified from database. A total of 1119 DEGs were identified from GSE35452 dataset, including 544 up-regulated genes and 575 down-regulated genes in READ cohort. [file 12967_2023_4029_MOESM1_ESM.pdf]

Identification of hub genes and potential molecular mechanisms related to radiotherapy sensitivity in rectal cancer based on multiple datasets

Pengfei Zhao[1], Hongchao Zhen[2], Hong Zhao[1], Yongjie Huang[1], Bangwei Cao[2#]

[1]Department of Radiotherapy, Beijing Friendship Hospital, Capital Medical University, Beijing 100050, P.R. China.

[2]Department of Oncology, Beijing Friendship Hospital, Capital Medical University, Beijing 100050, P.R. China.

Supplementary Figures and Legends

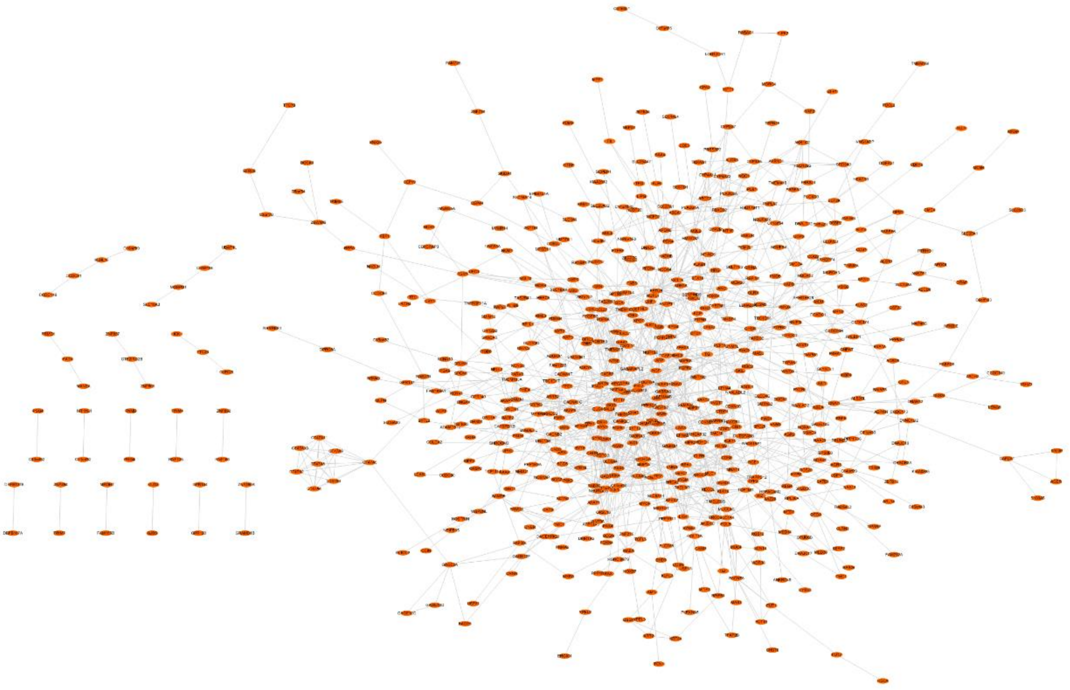

Supplementary Figure 1. Functional enrichment of DEGs by metascape enrichment analysis.

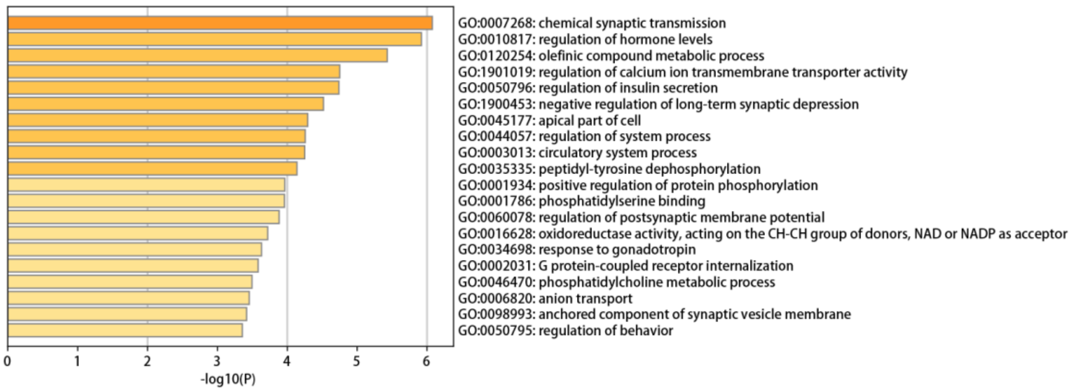

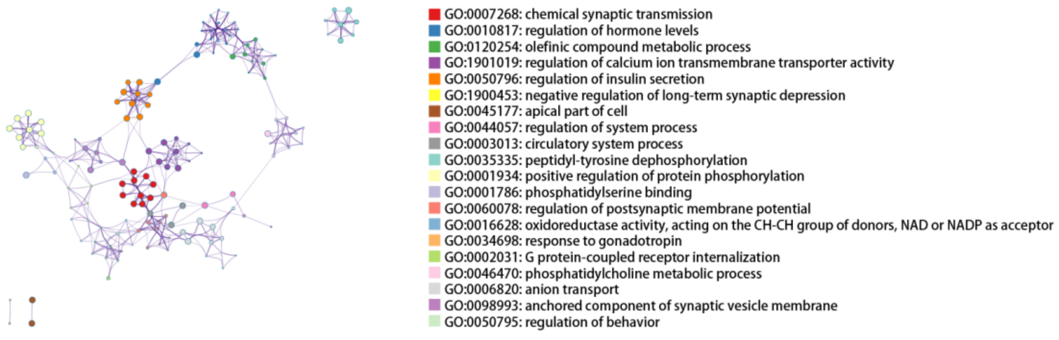

Supplementary Figure 2. Protein interaction network analysis of DEGs by Cytoscape

analysis. Circles represent genes, lines represent the interaction of proteins between genes.

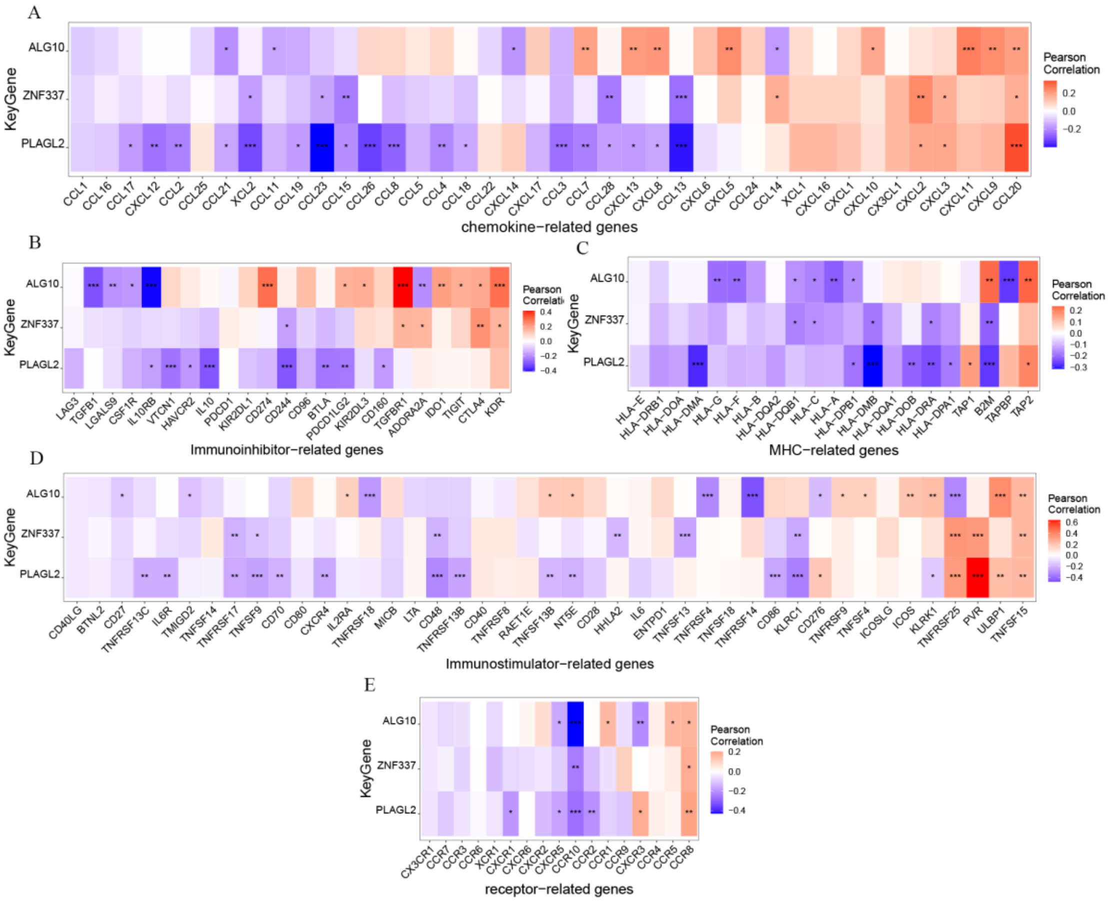

Supplementary Figure 3. The correlations between the expression of key genes (ALG 10, PLAGL2 and ZNF337) and different immune-related genes from the TISIDB database. A. Correlated with chemokines-related genes; B. Correlated with immunoinhibitor-related genes; C. Correlated with MHC-related genes; D. Correlated with immunostimulatory-related genes; E: Correlated with receptor-related genes. (* represented $P < 0.05$, ** represented $P < 0.01$ and *** represented $P < 0.001$; The redder the color, the stronger the positive correlation; The deeper of the purple color, the stronger the negative correlation)

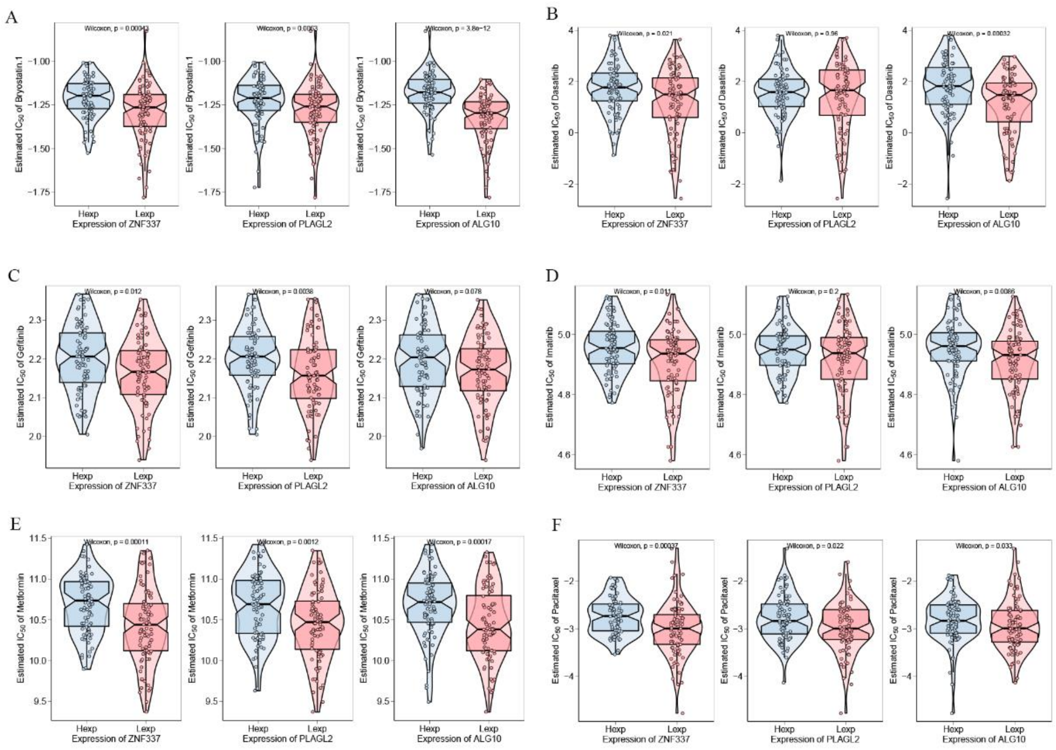

Supplementary Figure 4. The sensitivity analysis of chemotherapeutic drugs between different expression levels of key genes based on GDSC database. A: Bryostatin.1; B: Dasatinib; C: Gefitinib; D: Imatinib; E: Metformin; F: Paclitaxel.

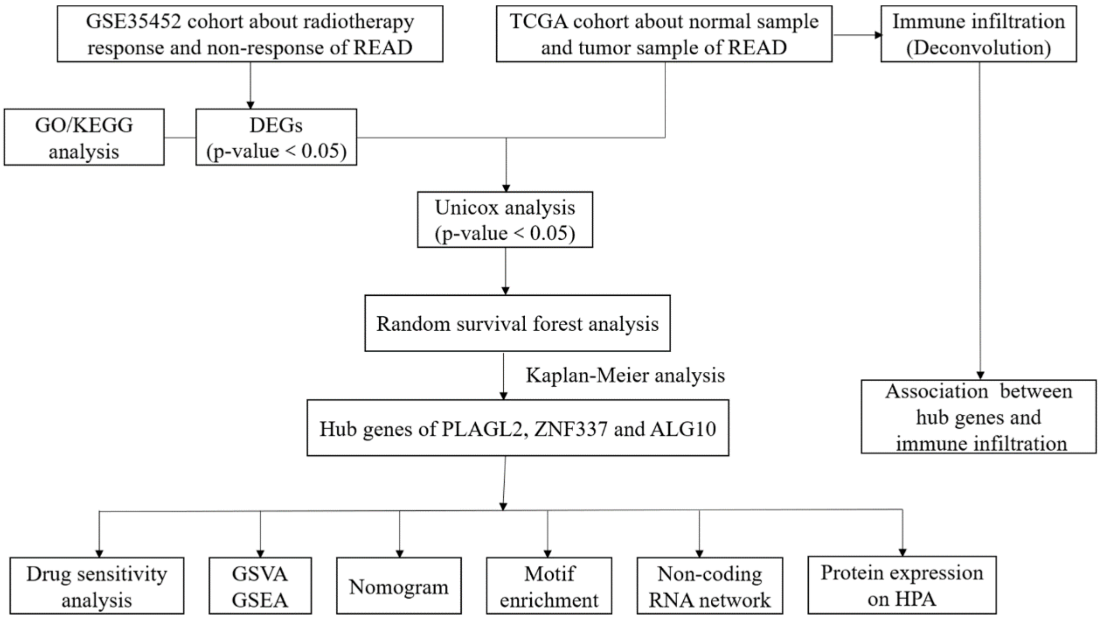

Supplementary Figure 5. The flowchart of the current study.

Supplemental Table

Supplementary Table 1. Information of the DEGs identified from database. A total of 1119 DEGs were identified from GSE35452 dataset, including 544 up-regulated genes and 575 down-regulated genes in READ cohort.

DEGs	Gene name
**Upregulated (544)**	LOC100131756, LRRIQ3, LOC101927151, BRCA2, RP11-495P10.9, RP3-522P13.1, UMODL1, C10orf62, MED14OS, LHX8, LOC101928401, SPACA3, DRD3, ENKUR, LOC101928288, CSMD2-AS1, RECQL, TMC1, TRA@, MTNR1B, SEMA3A, GABRA6, SPATC1, SNX25, STX16, RP11-171N4.1, UNC93A, LIG4, LOC284865, TRHR, MTCP1, B3GALTL, LOC100506538, LOC101926912, RP11-356B19.11, SLC6A4, LOC149373, C5orf64, PTPN11, LOC284939, LINC00424, C18orf15, RP11-585P4.5, LOC283075, USP12, GTF2IRD2B, NAA11, LINC01206, SYN1, C7orf25, DIAPH3, SCPEP1, RNF166, FAM230B, UQCC2, RP11-66N11.8, PALM, LINC00116, VAPB, GPR132, TRIM65, SLC26A4-AS1, KIR2DL2, FOXK1, LOC101929025, TPP2, LOC284798, CXCR3, OR51A10P, CMC4, RP11-1012E15.1, MANEA, RNF6, ANGPT1, KCNV1, CYP2A7, PFDN4, LINC00593, MTIF3, LOC729461, SPATC1L, MRGPRX4, LINC01348, PIPOX, CCND2, ZFP1, KLHL18, LINC00052, LINC01424, LINC00703, LOC101927988, MMP27, LOC101928545, YAF2, NRTN, RBM41, POLB, DOCK9, EDARADD, LOC101929289, CTB-78F1.1, LOC100271832, GPR107, GCNT4, GABRB1, PLB1, LINC01420, LOC101929709, HM13, LRRC37BP1, LOC101927650, TUSC8, AC064852.4, PPIAL4B, PPIAL4C, PPIAL4G, C14orf39, SNORD3A, SNORD3B-1, SNORD3B-2, SNORD3C, SNORD3D, LINC00323, DTD1, GRINA, FAM200A, UBD, LINC00461, MIR9-2, NAALAD2, SLC10A3, GS1-164F24.1, CXCL11, DEFB107A, DEFB107B, C10orf53, RP11-498C9.17, RFPL2, ADCY5, PHF2P1, LOC101929248, ZNF92, MED14, POU5F1, BC037833, RP11-492E3.2, LOC100652824, MIR3934, TCP11L1, FAM72A, FAM72B, FAM72C, FAM72D, SKIL, OR11A1, DDAH1, TMPRSS11B, EFR3B, PDGFRA, LOC101928435, LOC101927003, BC031255, RP11-260O18.1, NKAP, LOC101928446, ZNF280B, CYP4A11, CEP44, PARVB, RP11-360K13.1, CIDECP, AGER, MED30, BMP15, TGIF2LY, CXXC1P1, ABCB8, LOC285766, HRG, GPR143, MORC4, TCFL5, CASC2, POM121, CCDC116, RP11-402G3.5, TTC7B, LOC101927308, PTPRO, CEP19, AK054708, RP11-547D24.1, SGOL1, LOC646482, CTA-331P3.1, OGFRL1, LOC653786,

	OTOA, TIMM8A, MAST1, LDHAL6A, POMP, TMEM235, LOC101928850, SLC1A4, XRCC3, CTD-2313J17.5, HTR1B, LINC01153, RAP2C, PSG4, CPA2, LINC00592, REPIN1, TBC1D16, RAD54L2, TCP11, GRAP, AFP, GLRB, AL133493.2, NAGLU, LOC553103, LOC101928940, NXT2, LOC101930114, SALL4, LOC286121, MAFG-AS1, ZNF883, FAM71F1, PRKCQ, PARD3B, LOC101928259, LRRC58, FAM186B, LOC100505716, MPP1, PAN3-AS1, CRMP1, LINC01105, TG, LOC100287413, FAT4, LINC00305, PRIM2B, TTC26, NEURL3, AC005498.3, TFE3, SNHG17, GAGE12B, GAGE12F, GAGE12G, GAGE12I, GAGE4, GAGE5, GAGE6, GAGE7, MBL2, CT47A1, CT47A10, CT47A11, CT47A12, CT47A2, CT47A3, CT47A4, CT47A5, CT47A6, CT47A7, CT47A8, CT47A9, BAGE, HCG22, PLAGL2, GLB1L3, ADPRH, FUT1, USP31, FGFR1OP2, F8, ICK, LINC00189, KCNA10, HMGB1P4, ISY1-RAB43, LOC100506790, RP11-195M16.3, TRIM4, RPL36A, MRAP, PPP1R2, DNM1L, IRAK1, TMEM107, LOC101928555, SNAI1, GPR123, PLEKHA5, RP11-109D24.1, C8orf88, LOH12CR1, TMEM185A, RNU2-22P, LOC100130278, RP11-319E16.2, AGXT2, JPH3, DCAF10, MINA, GJD3, SART3, SYNE3, ZBTB12, ALG10, ZNF337, TSEN15, SYN3, LOC101928869, LOC101929662, SPIN3, LOC101060091, TAB3, FBRSL1, OR5P2, PYCRL, MAP4K5, LOC101927552, MAS1, LOC101926916, WDHD1, ZFR2, PRIM2, RPA1, LOC100506351, LOC440792, TIMELESS, SLC7A5, LINC00622, ATP6V1E2, RAX, IGSF22, ZNF738, ZNF275, SLCO4C1, SMG5, LOC101060264, SHROOM2, WWOX, GHSR, MIR4435-1HG, CYTH3, ZNF251, RP5-1085F17.3, RP1-192P9.1, ZMYND15, MORF4L2-AS1, CYP4Z1, FMR1, LCA5L, LINC00599, MIR124-1, LINC00330, RP11-305L7.3, ARRB2, LINC00535, PTPN1, BC113958, ADAMTS12, GSN-AS1, CAPZA1, TONSL, LINC00545, ZNF837, RNGTT, LOC101060424, NOL10, TMEM170B, FAM58A, RPS4XP9, FUT7, LOC285857, WBP4, NUP153, NOM1, BC044614, LOC101929734, FHL3, ITGA9, IRF8, MACROD2-AS1, SMIM12, DKFZp667F0711, LOC100128281, FBF1, FKSG29, HEATR9, ITGA9-AS1, SLC6A20, MGC15705, TRPM3, ZHX3, CNN2, CEP41, LOC102467079, NAA20, C6orf62, DNHD1, RP4-539M6.14, RP3-327A19.5, GPR78, LINC01186, ERVH-1, LOC101927138, APEX2, TEX41, BC027619, RP11-236J17.3, SLC25A32, COBLL1, FAM230C, DPM1, RFWD3, TMEM55A, ATP13A3, LOC101927820, LOC100505635, UBP1, TFDP3, OTUD6B, ZNF619, LOC100288490, CELP, SPC25, RP11-708J19.1, NFKBIE, CPSF2, C2orf81, RP11-960B9.2, RP11-

	248J18.3, CYP4A22, LAGE3, ACPT, ALG5, NUPL1, GABRR2, VN1R5, CHAT, ND6, MGC10814, INPP5J, HERC5, LINC01122, GAGE12C, GAGE12D, GAGE12E, GAGE12H, GUCY2D, GOLT1B, TAS2R41, RP11-180N14.1, RP11-649A18.7, SMTNL2, SKIDA1, KB-431C1.5, C3orf27, XPO4, C11orf58, NEDD1, LOC100506791, NAALADL2, LOC101927123, TMEM156, LOC101928230, LOC100422737, WDR4, FAM122B, C17orf75, GUSBP5, SLFNL1-AS1, RAD51AP1, DUSP13, ZXDB, KRT84, EHD3, DUSP21, LOC101928516, RP11-255C15.3, BC028670, CDKN1B, UBAP1L, FBXW4P1, ZNF429, LINC01103, GAGE2A, GAGE2C, DLEU2, AK093362, RP11-774D14.1, LOC101926907, GAS6-AS1, PCDHB5, DAOA-AS1, TEF, BATF2, MCFD2, RIBC2, SH2D3C, CELSR1, CARD18, PAQR3, RP11-324L17.1, BC028044, OR51A1P, RP11-329B9.5, TSR1, MGC39545, RP11-31K23.2, LAG3, TNMD, OR8D1, AC090627.1, LOC100506860, PNMA2, CTD-2286N8.2, NKPD1, STARD13-AS, LOC100128840, SLC6A5, TMSB15A, TOP1MT, EVA1A, RP11-806L2.2, XPOT, RCE1, SAA4, HGH1, RP11-549J18.1, LOC339568, MOSPD1, MME, APOOL, MNAT1, LINC00334
**Downregulated (575)**	S100A5, PTPRF, YPEL3, PDCL2, ARHGEF28, HSD17B11, C3orf70, LOC100506235, ADRB1, REG4, NPY6R, TMEM41B, PCF11, RP11-360A18.2, SEMA4B, COLCA2, EPHA4, RP11-363E7.4, PAX4, TGFBR3, DNAJC12, SERPINB5, TMEM26, 3-Mar, LOC101928417, SYT17, TRIM7, CD163L1, RP11-252E2.1, CTSE, PPP3CA, CLEC2L, ZADH2, IL1RAPL2, LOC93622, HSD17B2, MAGEC3, LINC01214, MIR6872, SPSB3, CD55, RAB27B, LOC101930241, CTB-113P19.1, KLKB1, LOC388780, PDK4, FRMD3, NOS1AP, TSPAN8, TRIM8, CDH19, AGPAT4-IT1, TRAV24, TMC7, MYBPC3, LOC101060405, PRG4, SMAD9, NOXA1, GDA, RHOBTB3, NME3, NGF, LINC01169, TNIK, ANKZF1, WDR38, CHGB, SRD5A2, CNGA4, ZNF488, LOC100129620, INSM1, EFNA5, ENO1-AS1, CYP3A5, BC036311, FEV, RP11-112J3.16, P4HA1, CATSPERB, DSG4, FETUB, LOC101928834, GATS, LRCH4, AK125860, RP11-582J16.4, ZNF302, OR10D1P, AGR3, PLA2G4A, LOC101930566, LOC102800447, MUC5AC, GABARAPL2, WISP3, LOC221272, BTBD10, MPC1, DNER, YTHDC1, OR2W1, C11orf52, HSPB2-C11orf52, RGS12, PDE9A, VSIG2, TLE1, ERVK13-1, SLC25A47, YPEL2, NOL3, SCG5, RP11-109M19.1, GAD1, NEUROG3, LOC284112, MIR195, MIR497, MIR497HG, STKLD1, CHST6, SKIV2L2, SPG11, LOC102724257, UNC13B, LOC101929172, NUDT22, FHL2, KIZ, SAMD5, ANXA2P2, HADHB, C19orf33, RASAL1,

	ARHGAP32, RFESD, SNORA37, PGM2L1, SSTR5-AS1, RP11-757F18.5, LOC101928099, GRAMD1B, SMCO4, OR2L13, CAPN12, CXorf30, SLC25A34, AX746710, OVOL1-AS1, KLK11, PCSK1, ABCC3, KCNMA1, NXF3, PAX6, MED7, QRICH1, CCIN, MYOF, UNC93B1, FAM181A-AS1, GMPR, MLPH, CYP2E1, PRUNE2, CTD-2311B13.7, P775P, KIAA1244, HMGCS2, ERGIC1, AP001062.7, CLEC4G, RP1L1, RANBP17, MIR4755, LOC644135, RAB3C, LINC00282, FGFR2, LOC100422781, U2AF1L4, MTCL1, DUSP4, LOC286359, CCDC174, SIDT2, ARL14, LOC101930052, LOC102724562, MST1P2, EEF2K, LOC101930123, YIPF1, LOC101927579, LOC101928042, AUH, NR1H4, NPW, PRKAG2-AS1, MC5R, OR7C2, C14orf180, TPH1, ACER3, CIB1, DHRS4-AS1, CCDC68, BAZ2B, LOC101930301, LOC101930303, TRIM16L, DMKN, LMF1, TAS2R16, NELL2, CACNA1C, TMEM167B, RP5-894A10.6, SSPO, LINC00261, C4BPB, AKAP7, RBM12B-AS1, CRYBA2, LOC101928519, PABPC5, KLK10, ERN2, SYT13, LOC654780, NQO1, INHBB, CDKN2D, DKK1, LOC153682, CACNB2, TMEM159, NT5E, CCDC90B, DLG4, AX747826, ALS2CL, NOSTRIN, RNF125, TFAP2B, MIR6824, C6orf163, GSN, TRAPPC12, SHC2, ZNF688, ZNF284, TMEM88, USP3-AS1, WNT9A, TSHZ1, CCDC140, RASSF6, ARHGEF4, MT1E, AJ606316, MROH7, PLLP, DMRTA2, LOC728084, IFNGR1, MTMR14, LOC100130417, PLA2G2A, HYPK, PLCXD3, ZBTB34, HOXD3, LOC401021, MIR1282, SERF2, KIAA0895, FAM155B, LOC102725213, TCEB1P3, IP6K2, C15orf43, MCU, LOC286382, CCDC60, B3GNT6, RSPO4, PHPT1, ARX, CHMP1B, ZSWIM6, WDR37, CAMK2D, GRIK4, MUC1, EFCAB4B, ANKRD49, DYNC1LI2, SMAD5-AS1, BAIAP3, SATB1, AC002310.12, AK056973, DUS3L, CX3CL1, HAVCR1, RARRES1, CBY1, C2orf72, INSR, CFC1, MKL2, CCDC153, LENG9, RP11-402D21.2, FAM222B, JMJD1C, NEDD4L, ACSM3, CCL27, CIB4, AQP7, LOC100509620, LOC101930168, LOC284661, FOXN4, SLC18B1, MBOAT2, LOC101928098, WISP2, C15orf52, RALBP1, CADPS2, KLHL3, LOC400620, MARVELD3, CCDC173, RP11-161D15.1, SCGN, NPTX1,,RP11-33O4.1, PTPRH, ACBD5, CAMP, LOC283737, DNAH12, AF086126, SLC35A1, LINC00265, ZNF692, FMO5, LINC00887, RP11-747H7.3, DYDC2, AKR1C1, ARHGAP36, CDSN, TSPAN1, CARD6, DNAI1, SLC30A10, MST1R, PPP1R12B, ZNF114, SPTSSB, TM4SF4, AKAP5, MYBPC1, CACNA1F, EPB41L4A, CTDP1, GCG, DNM1P35, SLC35D3, GABRA3, IRGM, RBFA, HECA, FLRT3, ZBTB7C, CDK5RAP3, FOXO4, SLC16A9,

	RABAC1, BIRC2, FAM179A, TIPARP, FLJ00290, MGLL, LINC00529, ITGB2-AS1, ZNF805, AGR2, HNRNPA1P37, CHGA, SLC5A9, C16orf89, ANG, NDUFS4, PTGER2, C11orf30, ARRDC2, CD1D, MGC27382, TBXAS1, ZFHX3, MB21D1, LINC00948, DNAJC4, FAM124B, TBC1D17, MUC4, TAGLN3, RGS20, OCEL1, LINC01007, APOA5, RBM43, PDE6H, VSIG10L, LOC101928837, LOC101927526, HGD, HEPACAM2, EPS8L3, MYO7B, TCN1, SEC14L4, ADH1A, SLC25A2, LOC101928676, MAEA, FUT10, LOC101927330, TNNT1, AY940074, VNN1, EFCAB4A, NBEA, JAG1, RP11-288L9.1, OSBP2, SPON1, ALAS2, LOC100506411, LOC100505592, SLC27A1, RP11-395B7.2, FAM160A2, TEC, ARAP1, SCG2, KCNG3, OR12D2, MAEL, ADAM32, PSG5, PPP6R3, TNFRSF11A, AP1G2, PPP1R11, SNORD23, RP6-24A23.7, ERCC6, PDXDC1, OBFC1, SYT8, SAP30L, ZNF180, CXorf58, NPIPB15, CRNN, UGP2, DUOXA2, CFD, ACTN3, RP11-710C12.1, C11orf80, LMNA, SLC12A6, TFG, FAM189A1, GNPAT, MAPK8, PTRHD1, AKR1C2, LOC101930400, HIPK2, SLC6A14,CLRN3, WBP2NL, CYSTM1, D21S2088E, PHEX, ZNF791, MXD3, IFNA1, DUOX1, CCDC81, CPS1-IT1, HMP19, LOC574538, MTMR9LP, BNIP3L, PNPLA7, AMN, SLC16A14, PPP1R36, KRT2, PPP1R9A, NPSR1-AS1, ITGB6, LOC100505984, FAM212A, DNAJC17, PP12708, SESTD1, LOC101930097, MFSD11, COQ2, MALAT1, SRPK3, BC047615, CDS1, CIRBP, FBXO32, AX747517, RAC3, NUDT12, MPRIP-AS1, SRD5A3, MPP2, SMC1B, GPR144, ELOVL3, HTR2B, GLRX, LINC00881, HSPA2, ADORA1, KBTBD3, C11orf54, FRMD1, RP11-254F7.1, BPIFB1, DNAJC16, ABI1, S100P, GPRC5A, SYTL2, IVNS1ABP, ABCA1, KCP, RP4-594L9.2, HOXB7, ALOX5, CDK6, CCDC37, NCOA4, UFC1, MT1HL1, YIPF2, PRSS2, HPCA, LIPG, GJB5, MATN4, SCNN1G, LOC101928583, PLEKHN1, SULT1C2, RAB26
